# Synthesis and Antioxidant Properties of Novel 1,2,3-Triazole-Containing Nitrones

**DOI:** 10.3390/antiox12010036

**Published:** 2022-12-24

**Authors:** Dimitra Hadjipavlou-Litina, Iwona E. Głowacka, José Marco-Contelles, Dorota G. Piotrowska

**Affiliations:** 1 Department of Pharmaceutical Chemistry, School of Pharmacy, Faculty of Health Sciences, Aristotle University of Thessaloniki, 54124 Thessaloniki, Greece; 2 Bioorganic Chemistry Laboratory, Faculty of Pharmacy, Medical University of Lodz, Muszyńskiego 1, 90-151 Lodz, Poland; 3 Laboratory of Medicinal Chemistry, Institute of Organic Chemistry (CSIC), Juan de la Cierva 3, 28006 Madrid, Spain; 4 Centre for Biomedical Network Research on Rare Diseases (CIBERER), CIBER, ISCIII, 46010 Madrid, Spain

**Keywords:** antioxidants, oxidative stress, 1,2,3-Triazole-containing nitrones

## Abstract

Herein, we report the synthesis and antioxidant capacity of twelve novel 1,2,3-triazole-containing nitrones such as *N*-(2-(4-aryl-1*H*-1,2,3-triazol-1-yl)ethylidene)methanamine oxides **8a**–**f** and *N*-(2-(4-aryl)-1*H*-1,2,3-triazol-1-yl)ethylidene)-2-methylpropan-2-amine oxides **9a**–**f**, bearing an *N*-methyl, and an *N-t*-butyl substituent, respectively, at the nitrogen of the nitrone motif. Nitrones **8** and **9** were studied with regard to their antioxidant ability, as well as their ability to inhibit soybean lypoxygenase (LOX), and their in vitro antioxidant activity. For this, we used three different antioxidant assays, such as that featuring the interaction with the water-soluble azo compound AAPH for the inhibition of lipid peroxidation (LP), the competition with the DMSO for scavenging hydroxyl radicals, and the ABTS^•+^–decolorization assay. *t*-Butyl nitrone **9e**, bearing the 2,4-difluorophenyl motif, showed a strong LP inhibitory effect (100%), close to the reference compound Trolox (93%), being the most potent LP inhibitor (LPi) of the whole series of tested nitrones. Nitrones **9d**, **9e** and **9f**, bearing the 4-fluorophenyl, 2,4-difluorophenyl, and 4-fluoro-3-methylphenyl motif, respectively, were almost equipotent, and the most potent hydroxyl radical scavengers (~100%), more potent than Trolox (88%), were used as a reference compound. Regarding the LOX inhibition, the most potent inhibitor was the *t*-butyl substituted nitrone **9f** (27 μM), bearing the 4-fluoro-3-methylphenyl motif, being 60-fold less potent than NDGA (0.45 μM), which was used as the standard in this test. The results from the antioxidant determination in the ABTS radical cation (ABTS^•+^) decolorization assay were not significant. *N*-Methyl nitrone **8f**, bearing the 4-fluoro-3-methylphenyl motif, was the only promising representative, with a value of 34.3%, followed by nitrone **9f** (16%). From the antioxidant analyses, we have identified *N*-(2-(4-(4-fluoro-3-methylphenyl)-1*H*-1,2,3-triazol-1-yl)ethylidene)-2-methylpropan-2-amine oxide (**9f**), bearing *t*-butyl and 4-fluoro-3-methylphenyl motifs in its structure, as the most balanced and potent antioxidant agent among the tested nitrones, as it was the most potent LOX inhibitor (27 μM), an extremely efficient and potent hydroxyl radical scavenger (99.9%), as well as one of the most potent LPi (87%) and ABTS^•+^ scavengers (16%).

## 1. Introduction

During oxidative stress (OS), an overproduction and accumulation of reactive oxygen species (ROS) or reactive nitrogen species (RNS) occur. As a consequence, the correct functioning of cells and tissues is altered [1]. OS is involved in the pathological mechanisms of some diseases, including arteriosclerosis, heart attacks, and strokes, as well as neurodegenerative disorders such as Parkinson’s and Alzheimer’s diseases. For this reason, the search for new and efficient protective agents against oxidative stress is urgent and of great importance.

**Figure 1:**
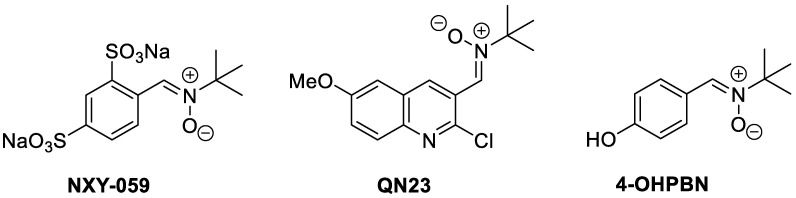
Examples of biologically active nitrones.

The role of both natural and synthetic antioxidants has received considerable attention during the past decades. Since nitrones are able to reduce oxidative stress by trapping ROS and RNS, they have been tested as potent antioxidants in various models of human diseases (Figure 1) [2]. For example, nitrone **NXY-059** has been recognized as an efficient neuroprotective agent in experimental studies [3] and reached clinical trials for the treatment of acute ischemic stroke [4]. While the antioxidant and neuroprotective properties of **QN23** have been proved [5], **4-OHPBN** was found to be an effective agent in the treatment of noise-induced hearing loss [6].

**Figure 2:**
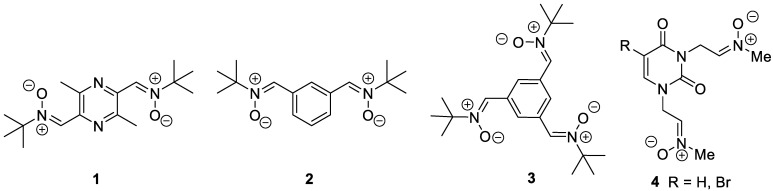
Examples of active bis- and tris-nitrones.

In the search for more active free radical scavengers, the idea of modifying the structure of known nitrones by incorporating additional nitrone functions or other structural units responsible for antioxidant potency has also been tested. Several bis- and tris-nitrones have been successfully designed (Figure 2). For example, bis-nitrones **1** [7] and **2** [8], and tris-nitrone **3** showed promising neuroprotective properties [9]. On the other hand, in the case of active nucleobase-containing nitrones, introducing an additional nitrone group did not result in a higher neuroprotective effect from the obtained bis-nitrones **4** compared to the respective mono-functionalized ones [10].

**Figure 3:**
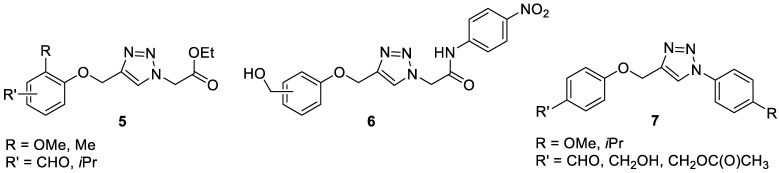
Examples of triazoles with antioxidant properties.

Increasing the potency of the antioxidant agent may also be achieved by combining two or even more structural and functional motifs with proven antioxidant activity. Having this general idea in mind, triazole moiety has attracted our attention, since several examples of antioxidants have been found in this class of compounds (Figure 3). And thus, 1,2,3-triazoles **5** exhibited moderate antioxidant activity (EC_50_ values above 75.5 µg/mL), and good log *P* values were determined for these compounds [11]. The antioxidant activity in the DPPH assay was noticed for compounds **6** [12] and **7** [13].

**Scheme 1:**
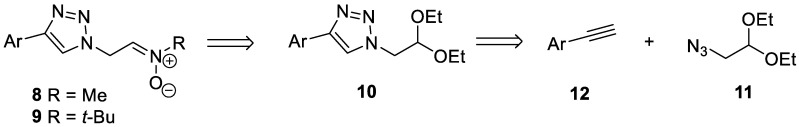
Retrosynthesis of 1,2,3-triazole-containing nitrones **8** and **9**.

The above-mentioned observations prompted us to design, synthesize and test the antioxidant properties of a series of 1,2,3-triazole-containing nitrones with general formulae **8** and **9** (Scheme 1). We reasoned that the synthesis of compounds **8** and **9** can be achieved by the application of, as a key step, Hüisgen cycloaddition of 2-azidoacetaldehyde diethyl acetate **11** with the respective alkynes **12**. The obtained Hüisgen cycloadducts **10** could be then easily hydrolyzed to the corresponding (1,2,3-triazole)aldehydes and subjected to the reaction with suitable and appropriate *N*-alkylhydroxylamines. From the antioxidant analyses, we have identified *N*-(2-(4-(4-fluoro-3-methylphenyl)-1*H*-1,2,3-triazol-1-yl)ethylidene)-2-methylpropan-2-amine oxide (**9f**), bearing *t*-butyl and 4-fluoro-3-methylphenyl motifs in its structure, as the most balanced and potent antioxidant agent among the tested nitrones.

## 2. Materials and Methods

### 2.1. Chemistry

#### 2.1.1. General Information

^1^H NMR spectra were taken in chloroform-d (CDCl_3_) or deuterium oxide (D_2_O) on a Bruker Avance III (600 MHz); ^13^C NMR spectra were recorded for CDCl_3_ solutions on the Bruker Avance III (600 MHz) spectrometer at 151 MHz. IR spectroscopic data were measured on a Bruker Alpha-T FT-IR spectrometer. Melting points were determined on a Boetius apparatus and were uncorrected. Elemental analyses were performed by the Microanalytical Laboratory of the Faculty of Pharmacy (Medical University of Lodz) on a Perkin Elmer PE 2400 CHNS analyzer and their results were found to be in good agreement (±0.3%) with the calculated values. All solvents were purified by the methods described in the literature.

#### 2.1.2. Synthesis of 2-Azidoacetaldehyde Diethyl Acetal **11**

A solution of 2-bromoethylacetaldehyde diethyl acetal in DMSO, NaN_3_ and KI were combined and the reaction mixture was stirred at rt for 15 min, and then at 90 °C for 5 d. After cooling down, water (15 mL) and diethyl ether (10 mL) were added, and the organic layer was separated. The aqueous phase was extracted with diethyl ether (4 × 15 mL). The combined organic extracts were dried over MgSO_4_, and then filtered and concentrated in vacuo to give pure azide **11** as colourless oil, in full agreement with literature data [14].

#### 2.1.3. General Procedure for the Synthesis of (1,2,3-Triazole)Acetaldehyde Diethyl Acetals **10a**–**f**

To a solution of azide **11** (1 mmol) in ethanol (1 mL) and water (1 mL), CuSO_4_ × H_2_O (0.1 mmol) and sodium ascorbate (0.05 mmol) were added, and these were followed by the respective alkyne **12a**–**f** (1 mmol). The suspension was microwave-irradiated in the Plazmatronika RM microwave reactor (30 W) at 40–45 °C for 1 h. After cooling, the solvent was removed in vacuo and the residue was suspended in chloroform (5 mL) and filtered through a layer of Celite. The obtained solution was concentrated in vacuo, and the crude product was chromatographed on a silica gel with a methylene chloride–methanol mixture (200:1, 100:1 and 50:1, *v*/*v*) to give the respective pure 1,2,3-triazole **10a**–**f**.

##### 1-(2,2-Diethoxyethyl)-4-Phenyl-1*H*-1,2,3-Triazole (**10a**)

Yield 94%; colourless oil; IR (film, cm^−1^) ν_max_ 3135, 3099, 3033, 2977, 2930, 2896, 1611, 1484, 1467, 1442, 1375, 1229, 1158, 1126, 1067; ^1^H NMR (200 MHz, CDCl_3_) δ 7.87–7.81 (m, 3H), 7.48–7.36 (m, 3H), 4.81 (t, *J* = 5.5 Hz, 1H), 4.50 (d, *J* = 5.4 Hz, 2H), 3.84–3.80 (m, 2H), 3.59–3.54 (m, 2H), 1.19 (t, *J* = 7.0 Hz, 6H); ^13^C NMR (151 MHz, CDCl_3_) δ 147.67, 130.69, 128.87, 128.13, 157.73, 121.06, 101.02, 63.94, 53.98, 15.24. Anal. Calcd. for C_14_H_19_N_3_O_2_: C, 64.35; H, 7.33; N, 16.08. Found: C, 64.53; H, 7.67; N, 15.95.

##### 1-(2,2-Diethoxyethyl)-4-(2-Fluorophenyl)-1*H*-1,2,3-Triazole (**10b**)

Yield 89%; colourless oil; IR (film, cm^−1^) ν_max_ 3154, 3072, 2978, 2931, 2897, 1804, 1746, 1583, 1557, 1487, 1376, 1234, 1220, 1126, 1072; ^1^H NMR (600 MHz, CDCl_3_) δ 8.31–8.27 (m, 1H), 8.05 (d, *J* = 3.8 Hz, 1H), 7.32–7.28 (m, 1H), 7.27–7.23 (m, 2H), 7.16–7.12 (m, 1H), 4.81 (t, *J* = 5.5 Hz, 1H), 4.51 (d, *J* = 5.5 Hz, 2H), 3.79–3.73 (m, 2H), 3.53–3.47 (m, 2H), 1.19 (t, *J* = 7.1 Hz, 6H); ^13^C NMR (151 MHz, CDCl_3_) δ 159.25 (d, *J* = 247.5 Hz), 141.07 (d, *J* = 2.1 Hz), 129.25 (d, *J* = 7.8 Hz), 127.80 (d, *J* = 3.3 Hz), 124.59 (d, *J* = 3.3 Hz), 124.21 (d, *J* = 13.1 Hz), 118.68 (d, *J* = 12.1 Hz), 115.69 (d, *J* = 22.1 Hz), 100.97, 63.88, 52.92, 15.19. Anal. Calcd. for C_14_H_18_N_3_O_2_F: C, 60.20; H, 6.50; N, 15.04. Found: C, 60.53; H, 6.72; N, 14.82.

##### 1-(2,2-Diethoxyethyl)-4-(3-Fluorophenyl)-1*H*-1,2,3-Triazole (**10c**)

Yield 80%; colourless oil; IR (film, cm^−1^) ν_max_ 3405, 3138, 2978, 2932, 2897, 1788, 1750, 1620, 1590, 1485, 1468, 1447, 1376, 1153, 1127, 1069; ^1^H NMR (600 MHz, CDCl_3_) δ 7.88 (s, 1H), 7.61–7.58 (m, 1H), 7.57–7.54 (m, 1H), 7.41–7.37 (m, 1H), 7.05–7.02 (m, 1H), 4.80 (t, *J* = 5.4 Hz, 1H), 4.50 (d, *J* = 5.3 Hz, 2H), 3.79–3.73 (m, 2H), 3.54–3.49 (m, 2H), 1.19 (t, *J* = 7.0 Hz, 6H); ^13^C NMR (151 MHz, CDCl_3_) δ 163.20 (d, *J* = 245.4 Hz), 146.58, 132.85 (d, *J* = 8.9 Hz), 130.44 (d, *J* = 7.7 Hz), 121.45, 121.30 (d, *J* = 2.1 Hz), 114.91 (d, *J* = 21.4 Hz), 112.64 (d, *J* = 23.1 Hz), 100.87, 63.92, 52.97, 15.23. Anal. Calcd. For C_14_H_18_N_3_O_2_F: C, 60.20; H, 6.50; N, 15.04. Found: C, 60.14; H, 6.67; N, 15.00.

##### 1-(2,2-Diethoxyethyl)-4-(4-Fluorophenyl)-1*H*-1,2,3-Triazole (**10d**)

Yield 90%; colourless oil; IR (film, cm^−1^) ν_max_ 3140, 2979, 2897, 1801, 1612, 1561, 1498, 1459, 1228, 1157, 1127, 1069; ^1^H NMR (200 MHz, CDCl_3_) δ 7.84–7.76 (m, 3H), 7.16–7.07 (m, 2H), 4.80 (t, *J* = 5.4 Hz, 1H), 4.49 (d, *J* = 5.3 Hz, 2H), 3.80–3.69 (m, 2H), 3.59–3.47 (m, 2H), 1.19 (t, *J* = 7.0 Hz, 6H); ^13^C NMR (151 MHz, CDCl_3_) δ 162.66 (d, *J* = 246.6 Hz), 146.80, 127.44 (d, *J* = 8.5 Hz), 126.95 (d, *J* = 3.3 Hz), 120.78, 115.81 (d, *J* = 21.9 Hz), 100.92, 63.85, 52.95, 15.21. Anal. Calcd. For C_14_H_18_N_3_O_2_F: C, 60.20; H, 6.50; N, 15.04. Found: C, 60.33; H, 6.62; N, 15.17.

##### 1-(2,2-Diethoxyethyl)-4-(2,4-Difluorophenyl)-1*H*-1,2,3-Triazole (**10e**)

Yield 91%; colourless oil; IR (film, cm^−1^) ν_max_ 3424, 3157, 2976, 2928, 1800, 1625, 1601, 1494, 1418, 1145, 1130, 1053; ^1^H NMR (600 MHz, CDCl_3_) δ 8.29–8.24 (m, 1H), 8.00 (d, *J* = 3.8 Hz, 1H), 7.00–6.99 (m, 1H), 6.92–6.88 (m, 1H), 4.80 (t, *J* = 5.4 Hz, 1H), 4.51 (d, *J* = 5.4 Hz, 2H), 3.79–3.73 (m, 2H), 3.54–3.48 (m, 2H), 1.19 (t, *J* = 7.0 Hz, 6H); ^13^C NMR (151 MHz, CDCl_3_) δ 162.53 (dd, *J* = 250.2 Hz, *J* = 12.2 Hz), 159.29 (dd, *J* = 250.9 Hz, *J* = 12.1 Hz), 140.43, 128.86 (dd, *J* = 9.8 Hz, *J* = 5.4 Hz), 123.68 (d, *J* = 12.6 Hz), 115.19 (dd, *J* = 13.3 Hz, *J* = 4.3 Hz), 111.96 (dd, *J* = 21.0 Hz, *J* = 3.4 Hz), 104.08 (dd, *J* = 25.3 Hz, *J* = 26.4 Hz), 100.90, 63.28, 52.94, 15.16. Anal. Calcd. for C_14_H_17_N_3_O_2_F_2_: C, 56.56; H, 5.76; N, 14.03. Found: C, 56.65; H, 5.48; N, 13.92.

##### 1-(2,2-Diethoxyethyl)-4-(4-Fluoro-3-Methylphenyl)-1*H*-1,2,3-Triazole (**10f**)

Yield 80%; colourless oil; IR (film, cm^−1^) ν_max_ 3355, 3136, 2978, 2930, 2896, 1804, 1557, 1495, 1459, 1120, 1061; ^1^H NMR (200 MHz, CDCl_3_) δ 7.80 (s, 1H), 7.72–7.66 (m, 1H), 7.62–7.53 (m, 1H), 7.10–7.00 (m, 1H), 7.72–7.66 (m, 1H), 4.80 (t, *J* = 5.4 Hz, 1H), 4.48 (d, *J* = 5.3 Hz, 2H), 3.84–3.68 (m, 2H), 3.56–3.43 (m, 2H), 2.32 (d, *J* = 1.9 Hz, 3H), 1.19 (t, *J* = 7.0 Hz, 6H); ^13^C NMR (151 MHz, CDCl_3_) δ 161.24 (d, *J* = 245.5 Hz), 146.98, 128.87 (d, *J* = 5.5 Hz), 126.52 (d, *J* = 3.2 Hz), 125.39 (d, *J* = 17.6 Hz), 124.72 (d, *J* = 15.2 Hz), 120.74, 115.42 (d, *J* = 23.1 Hz), 100.96, 63.91, 52.95, 15.23, 14.61 (d, *J* = 3.3 Hz). Anal. Calcd. for C_15_H_20_N_3_O_2_F: C, 61.42; H, 6.87; N, 14.32. Found: C, 61.35; H, 7.01; N, 14.09.

#### 2.1.4. General Procedure for the Synthesis of Nitrones **8a**–**f** and **9a**–**f**

A solution of the respective diethyl acetal **10a**–**f** (0.1 mmol) in 1M HCl (1 mL) was stirred at reflux for 1 h. After that, the solvent was removed and the residue re-evaporated with water until a neutral pH was obtained. The obtained aldehyde **13a**–**f** was immediately used in the next step without further purification. To a solution of the obtained aldehyde **13a**–**f** in ethanol (2 mL), CH_3_CO_2_Na (1.3 mmol) and *N*-alkyhydoxylamine hydrochloride (1.1 mmol) were added. The reaction mixture was stirred until the disappearance of the starting aldehyde was noticed on TLC. After that 10% NaHCO_3_ was added (5 mL) and the product was extracted with methylene chloride (3 × 5 mL). Organic extracts were combined, dried (MgSO_4_), concentrated, and crystallized to give the respective pure nitrone **8a**–**f** or **9a**–**f**.

##### *N*-(2-(4-Phenyl-1*H*-1,2,3-Triazol-1-yl)Ethylidene)Methanamine Oxide (**8a**)

Yield 69%; white amorphous solid; mp 97–8 °C (recrystallized from methylene chloride—ethyl ether); IR (KBr, cm^−1^) ν_max_ 3406, 3115, 3084, 3060, 2964, 1614, 1484, 1462, 1437, 1225, 1204, 1148, 1077, 1048; ^1^H NMR (600 MHz, CDCl_3_) δ 8.00 (s, 1H), 7.84–7.81 (m, 2H), 7.44–7.41 (m, 2H), 7.36–7.33 (m, 1H), 7.18–7.17 (m, 1H), 5.39–5.36 (m, 2H), 3.79 (d, *J* = 0.7 Hz, 3H); ^13^C NMR (151 MHz, CDCl_3_) δ 148.16, 131.93, 130.29, 128.88, 128.34, 125.80, 121.01, 52.76, 45.71. Anal. Calcd. for C_11_H_12_N_4_O: C, 61.10; H, 5.59; N, 25.92. Found: C, 60.91; H, 5.51; N, 25.70.

##### *N*-(2-(4-(2-Fluorophenyl)-1*H*-1,2,3-Triazol-1-yl)Ethylidene)Methanamine Oxide (**8b**)

Yield 63%; white amorphous solid; mp 125–6 °C (recrystallized from methylene chloride—ethyl ether); IR (KBr, cm^−1^) ν_max_ 3425, 3176, 3094, 3060, 3006, 1617, 1583, 1556, 1488, 1469, 1425, 1393, 1318, 1239, 1213, 1073; ^1^H NMR (600 MHz, CDCl_3_) δ 8.29–8.25 (m, 1H), 8.11 (d, *J* = 3.6 Hz, 1H), 7.33–7.30 (m, 1H), 7.27–7.23 (m, 1H), 7.18–7.12 (m, 2H), 5.42–5.40 (m, 2H), 3.79 (d, *J* = 0.8 Hz, 3H); ^13^C NMR (151 MHz, CDCl_3_) δ 159.29 (d, *J* = 248.6 Hz), 141.60 (d, *J* = 2.1 Hz), 132.04, 129.53 (d, *J* = 8.6 Hz), 127.84 (d, *J* = 3.5 Hz), 124.61 (d, *J* = 3.3 Hz), 123.93 (d, *J* = 12.5 Hz), 118.30 (d, *J* = 12.9 Hz), 115.73 (d, *J* = 21.8 Hz), 52.70, 46.02. Anal. Calcd. for C_11_H_11_N_4_OF: C, 56.41; H, 4.73; N, 23.92. Found: C, 56.73; H, 4.56; N, 24.11.

##### *N*-(2-(4-(3-Fluorophenyl)-1*H*-1,2,3-Triazol-1-yl)Ethylidene)Methanamine Oxide (**8c**)

Yield 61%; white amorphous solid; mp 158–160 °C (recrystallized from methylene chloride—ethyl ether); IR (KBr, cm^−1^) ν_max_ 3424, 3133, 3095, 2871, 1621, 1589, 1561, 1484, 1229, 1181; ^1^H NMR (600 MHz, D_2_O) δ 8.31 (s, 1H), 7.55–7.50 (m, 2H), 7.47–7.44 (m, 1H), 7.43–7.39 (m, 1H), 7.10–7.07 (m, 1H), 5.38–5.34 (d, *J* = 4.2 Hz, 2H), 3.69 (d, *J* = 0.7 Hz, 3H); ^13^C NMR (151 MHz, CDCl_3_) δ 163.22 (d, *J* = 246.2 Hz), 147.05, 132.48 (d, *J* = 9.2 Hz), 131.54, 130.46 (d, *J* = 8.1 Hz), 121.45**,** 121.40 (d, *J* = 2.9 Hz), 115.15 (d, *J* = 21.0 Hz), 112.75 (d, *J* = 23.0 Hz), 52.83, 45.63. Anal. Calcd. For C_11_H_11_N_4_OF: C, 56.41; H, 4.73; N, 23.92. Found: C, 56.26; H, 4.43; N, 23.96.

##### *N*-(2-(4-(4-Fluorophenyl)-1*H*-1,2,3-Triazol-1-yl)Ethylidene)Methanamine Oxide (**8d**)

Yield 78%; white amorphous solid; mp 165–7 °C (recrystallized from methylene chloride—ethyl ether); IR (KBr, cm^−1^) ν_max_ 3363, 3203, 3134, 2877, 2382, 1614, 1562, 1499, 1458, 1238, 1158, 1093; ^1^H NMR (600 MHz, D_2_O) δ 8.33 (s, 1H), 7.81–7.78 (m, 2H), 7.63–7.61 (m, 1H), 7.26–7.22 (m, 2H), 5,47–5.42 (m, 2H), 3.78 (s, 3H); ^13^C NMR (151 MHz, CDCl_3_) δ 162.80 (d, *J* = 247.6 Hz), 147.27, 131.68, 127.54 (d, *J* = 7.8 Hz), 126.54 (d, *J* = 3.3 Hz), 120.81, 115.87 (d, *J* = 21.8 Hz), 52.81, 45.61. Anal. Calcd. for C_11_H_11_N_4_OF: C, 56.41; H, 4.73; N, 23.92. Found: C, 56.52; H, 4.82; N, 23.83.

##### *N*-(2-(4-(2,4-Difluorophenyl)-1*H*-1,2,3-Triazol-1-yl)Ethylidene)Methanamine Oxide (**8e**)

Yield 56%; white amorphous solid; mp 122–3 °C (recrystallized from methylene chloride—ethyl ether); IR (KBr, cm^−1^) ν_max_ 3424, 3179, 3094, 3058, 1619, 1599, 1459, 1412, 1397, 1268, 1126, 1067; ^1^H NMR (600 MHz, D_2_O) δ 8.36 (d, *J* = 2.9 Hz, 1H), 7.99–7.94 (m, 1H), 7.63 (t, *J* = 5.0 Hz, 1H), 7.13–7.08 (m, 2H), 5.47 (d, *J* = 4.8 Hz, 2H), 3.78 (s, 3H); ^13^C NMR (151 MHz, CDCl_3_) δ 162.69 (dd, *J* = 250.8 Hz, *J* = 13.1 Hz), 159.33 (dd, *J* = 250.9 Hz, *J* = 12.2 Hz), 140.92 (d, *J* = 2.8 Hz), 131.73, 128.82 (dd, *J* = 9.8 Hz, *J* = 5.4 Hz), 123.48 (d, *J* = 11.9 Hz), 114.79 (dd, *J* = 13.1 Hz, *J* = 3.2 Hz), 112.03 (dd, *J* = 22.0 Hz, *J* = 3.5 Hz), 104.16 (dd, *J* = 26.4 Hz, *J* = 25.2 Hz), 52.76, 45.93. Anal. Calcd. for C_11_H_10_N_4_OF_2_ × 0.25H_2_O: C, 51.46; H, 4.12; N, 21.83. Found: C, 51.60; H, 3.85; N, 21.74.

##### *N*-(2-(4-(4-Fluoro-3-Methylphenyl)-1*H*-1,2,3-Triazol-1-yl)Ethylidene)Methanamine Oxide (**8f**)

Yield 61%; white amorphous solid; mp 171–3 °C (recrystallized from methylene chloride—ethyl ether); IR (KBr, cm^−1^) ν_max_ 3171, 3130, 2882, 159, 1448, 1461, 1237, 1211, 1168, 1121; ^1^H NMR (600 MHz, D_2_O) δ 8.20 (s, 1H), 7.57–7.48 (m, 3H), 7.08–7.05 (m, 1H), 5,35–5.33 (d, *J* = 5.2 Hz, 2H), 3.69 (s, 3H), 2.20 (s, 3H); ^13^C NMR (151 MHz, CDCl_3_) δ 161.36 (d, *J* = 246.5 Hz), 147.46, 131.87, 128.95 (d, *J* = 5.6 Hz), 126.11 (d, *J* = 4.3 Hz), 125.47 (d, *J* = 17.6 Hz), 124.78 (d, *J* = 7.8 Hz), 120.77, 115.48 (d, *J* = 23.05 Hz), 52.82, 45.64, 14.58 (d, *J* = 3.3 Hz). Anal. Calcd. for C_12_H_13_N_4_OF: C, 58.06; H, 5.28; N, 22.57. Found: C, 57.78; H, 4.99; N, 22.61.

##### 2-Methyl-*N*-(2-(4-Phenyl-1*H*-1,2,3-Triazol-1-yl)Ethylidene)Propan-2-Amine Oxide (**9a**)

Yield 85%; white amorphous solid; mp 91–3 °C (recrystallized from methylene chloride—ethyl ether); IR (KBr, cm^−1^) ν_max_ 3375, 3328, 3134, 2973, 2872, 1611, 1470, 1442, 1363, 1118, 1085, 1052, 1001; ^1^H NMR (600 MHz, CDCl_3_) δ 7.98 (s, 1H), 7.86–7.81 (m, 2H), 7.48–7.29 (m, 4H), 5.39 (d, 2H, *J* = 5.4 Hz), 1.53 (s, 9H); ^13^C NMR (151 MHz, CDCl_3_): 148.12, 130.39, 128.86, 128.29, 127.21, 125.80, 120.94, 70.68, 46.73, 27.87. Anal. Calcd. for C_14_H_18_N_4_O: C, 65.09; H, 7.02; N, 21.69. Found: C, 64.93; H, 6.87; N, 21.96.

##### *N*-(2-(4-(2-Fluorophenyl)-1*H*-1,2,3-Triazol-1-yl)Ethylidene)-2-Methylpropan-2-Amine Oxide (**9b**)

Yield 39%; white amorphous solid; mp 72–3 °C (recrystallized from ethyl ether—petroleum ether); IR (KBr, cm^−1^) ν_max_ 3417, 3137, 2974, 1625, 1583, 1488, 1390, 1220, 1109, 1076; ^1^H NMR (600 MHz, CDCl_3_) δ 8.28–8.25 (m, 1H), 8.09 (d, *J* = 3.6 Hz, 1H), 7.33–7.20 (m, 3H), 7.15–7.11 (m, 1H), 5.40 (d, *J* = 4.9 Hz, 2H), 1.52 (s, 9H); ^13^C NMR (151 MHz, CDCl_3_) δ 159.29 (d, *J* = 247.5 Hz), 141.55 (d, *J* = 3.3 Hz), 129.48 (d, *J* = 7.8 Hz), 127.83 (d, *J* = 4.0 Hz), 127.43, 124.60 (d, *J* = 3.3 Hz), 123.90 (d, *J* = 12.8 Hz), 118.37 (d, *J* = 12.5 Hz), 115.70 (d, *J* = 22.0 Hz), 70.62, 47.05, 27.85. Anal. Calcd. For C_14_H_17_N_4_OF×0.5H_2_O: C, 58.93; H, 6.36; N, 19.64. Found: C, 58.99; H, 6.49; N, 19.51.

##### *N*-(2-(4-(3-Fluorophenyl)-1*H*-1,2,3-Triazol-1-yl)Ethylidene)-2-Methylpropan-2-Amine Oxide (**9c**)

Yield 30%; white amorphous solid; mp 94–5 °C (recrystallized from methylene chloride—ethyl ether); IR (KBr, cm^−1^) ν_max_ 3417, 3127, 2975, 1620, 1589, 1485, 1460, 1392, 1364, 1227; ^1^H NMR (600 MHz, CDCl_3_) δ 8.00 (s, 1H), 7.60–7.55 (m, 2H), 7.41–7.37 (m, 1H), 7.30–7.28 (m, 1H), 7.05–7.02 (m, 1H), 5.38 (d, *J* = 5.2 Hz, 2H), 1.53 (s, 9H); ^13^C NMR (151 MHz, CDCl_3_) δ 163.21 (d, *J* = 245.6 Hz), 146.99 (d, *J* = 2.7 Hz), 132.58 (d, *J* = 8.4 Hz), 130.44 (d, *J* = 8.6 Hz), 126.86, 121.38, 121.38, 115.08 (d, *J* = 21.3 Hz), 112.73 (d, *J* = 23.1 Hz), 70.76, 46.64, 27.87. Anal. Calcd. For C_14_H_17_N_4_OF×1.5H_2_O: C, 55.43; H, 6.65; N, 18.48. Found: C, 55.16; H, 6.63; N, 18.36.

##### *N*-(2-(4-(4-Fluorophenyl)-1*H*-1,2,3-Triazol-1-yl)Ethylidene)-2-Methylpropan-2-Amine Oxide (**9d**)

Yield 37%; white amorphous solid; mp 128–131 °C (recrystallized from ethyl ether); IR (KBr, cm^−1^) ν_max_ 3425, 2975, 2533, 1613, 1562, 1499, 1227, 1158; ^1^H NMR (600 MHz, CDCl_3_) δ 7.95 (s, 1H), 7.82–7.79 (m, 2H), 7.30–7.27 (m, 1H), 7.14–7.11 (m, 2H), 5.38 (d, *J* = 5.2 Hz, 2H), 1.53 (s, 9H); ^13^C NMR (151 MHz, CDCl_3_) δ 162.78 (d, *J* = 247.6 Hz), 147.23, 127.53 (d, *J* = 7.6 Hz), 126.98, 126.63 (d, *J* = 3.6 Hz), 120.73, 115.86 (d, *J* = 22.1 Hz), 70.73, 46.64, 27.88. Anal. Calcd. for C_14_H_17_N_4_OF×0.5H_2_O: C, 58.93; H, 6.36; N, 19.64. Found: C, 58.94; H, 6.17; N, 19.85.

##### *N*-(2-(4-(2,4-Difluorophenyl)-1*H*-1,2,3-Triazol-1-yl)Ethylidene)-2-Methylpropan-2-Amine Oxide (**9e**)

Yield 43%; white amorphous solid; mp 122–3 °C (recrystallized from ethyl ether); IR (KBr, cm^−1^) ν_max_ 3425, 3167, 2923, 1629, 1599, 1562, 1396, 1211, 1131, 1073; ^1^H NMR (600 MHz, CDCl_3_) δ 8.29–8.24 (m, 1H), 8.06 (d, *J* = 3.6 Hz, 1H), 7.28–7.26 (m, 1H), 7.02–6.98 (m, 1H), 6.93–6.88 (m, 1H), 5.40 (d, *J* = 5.0 Hz, 2H), 1.53 (s, 9H); ^13^C NMR (151 MHz, CDCl_3_) δ 162.67 (dd, *J* = 249.8 Hz, *J* = 12.1 Hz), 159.32 (dd, *J* = 251.3 Hz, *J* = 10.9 Hz), 140.88 (d, *J* = 2.7 Hz), 128.84 (dd, *J* = 9.2 Hz, *J* = 4.7 Hz), 127.13, 123.44 (d, *J* = 12.2 Hz), 114.86 (d, *J* = 13.2 Hz), 112.03 (dd, *J* = 21.1 Hz, *J* = 3.2 Hz), 104.16 (dd, *J* = 25.3 Hz, *J* = 25.4 Hz), 70.66, 46.99, 27.86. Anal. Calcd. for C_14_H_16_N_4_OF_2_×0.25H_2_O: C, 56.27; H, 5.57; N, 18.76. Found: C, 56.56; H, 5.41; N, 18.68.

##### *N*-(2-(4-(4-Fluoro-3-Methylphenyl)-1*H*-1,2,3-Triazol-1-yl)Ethylidene)-2-Methylpropan-2-Amine Oxide (**9f**)

Yield 78%; white amorphous solid; mp 104–7 °C (recrystallized from ethyl ether); IR (KBr, cm^−1^) ν_max_ 3406, 3128, 3100, 2978, 1495, 1468, 1393, 1309, 1281, 1209, 1082; ^1^H NMR (600 MHz, D_2_O) δ 7.92 (s, 1H), 7.70–7.68 (m, 1H), 7.59–7.57 (m, 1H), 7.29–7.27 (m, 1H), 7.07–7.03 (m, 1H), 5.37 (d, *J* = 5.2 Hz, 2H), 2.32 (d, *J* = 1.8 Hz, 3H), 1.53 (s, 9H); ^13^C NMR (151 MHz, CDCl_3_) δ 161.35 (d, *J* = 246.5 Hz), 147.42, 128.95 (d, *J* = 5.6 Hz), 127.16, 126.23 (d, *J* = 3.3 Hz), 125.44 (d, *J* = 18.1 Hz), 124.79 (d, *J* = 7.7 Hz), 120.67, 115.45 (d, *J* = 23.2 Hz), 70.70, 46.67, 27.87, 14.55 (d, *J* = 3.3 Hz). Anal. Calcd. for C_15_H_19_N_4_OF×0.25H_2_O: C, 61.10; H, 6.67; N, 19.01. Found: C, 60.80; H, 6.70; N, 19.29.

### 2.2. Estimation of Lipophilicity as Clog P

We used Bioloom of Biobyte Corp for the theoretical calculation of lipophilicity as Clog *P* values (BioByte Home Page. Available online: http://www.biobyte.com, accessed on 22 November 2022).

### 2.3. Antioxidant Assays

The in vitro antioxidant assays for nitrones **8** and **9** were performed concentrations of 100 µM (from a stock solution 10 mM in 0.1% DMSO in deionized water). Several dilutions were made when the determination of IC_50_ values was needed. All the determinations were made, at least in triplicate, and the standard deviation of absorbance was less than 10% of the mean. The nitrones were diluted under sonification in the appropriate buffer in several dilutions (Table 1).

The following assays were used: (*i*) the ILP induced by AAPH, (*ii*) the competition of the tested nitrones with DMSO for hydroxyl radicals, (*iii*) the ABTS^+·^–decolorization assay, and (*iv*) the in vitro inhibition of soybean LOX.

#### 2.3.1. Materials and Methods

NDGA, Trolox, AAPH, soybean LOX, and linoleic acid sodium salt were purchased from Aldrich Chemical Co. (Milwaukee, WI, USA). Phosphate buffer (0.1 M, pH 7.4) was prepared by mixing an aqueous KH_2_PO_4_ solution (50 mL, 0.2 M), and an aqueous NaOH solution (78 mL, 0.1 M); 2-Amino-2-hydroxymethyl-propane-1,3-diol (Tris) was used as a buffer pH 9. A lambda 20 (Perkin–Elmer-PharmaSpec 1700, Perkin-Elmer Corporation Ltd., Lane Beaconsfield, Bucks, UK) UV–Vis double beam spectrophotometer was used for the assays.

To measure in vitro antioxidant activity of the nitrones **8** and **9**, the following assays were used: The inhibition of lipid peroxidation (LP) induced by AAPH, the DMSO method for hydroxyl radical scavenging activity, the ABTS^+·^–decolorization assay and the in vitro inhibition of soybean lipoxygenase (LOX).

#### 2.3.2. Inhibition of Linoleic Acid Peroxidation

The production of conjugated diene hydroperoxide by the oxidation of linoleate sodium 16 mM linoleate sodium (10 µL) in an aqueous solution is monitored at 234 nm. AAPH 40 mM (50 µL) is used as a free radical initiator at 37 °C under air conditions, followed by the tested nitrones. This assay can be used to follow oxidative changes by recording the absorbance values at 234 nm, using Trolox as a reference compound. The experimental procedure follows our previously reported protocol [15].

#### 2.3.3. In Vitro Inhibition of Soybean Lipoxygenase (LOX)

The in vitro study was evaluated as reported previously [15,16]. Compounds **8a**–**f** or **9a**–**f** (10 μL) were incubated at rt with sodium linoleate (0.1 mM) and 200 μL of enzyme solution (1/9 × 10^–4^ *w*/*v* in saline). Tris buffer pH 9 was inserted. The conversion of sodium linoleate to 13-hydroperoxylinoleic acid was recorded at 234 nm. NDGA was used as a positive control (IC_50_ = 0.45 µM or 87% at 100 µM). Several dilutions of compounds were used for the determination of IC_50_ values. Blank determination served as the negative control.

#### 2.3.4. Competition of Nitrones **8** and **9** with DMSO for Hydroxyl Radicals

The hydroxyl radicals, produced by the Fe^3+^/ascorbic acid system, were detected by the determination of formaldehyde produced from the oxidation of DMSO [8]. Solutions of EDTA (0.1 mM), Fe^3+^ (167 μM), DMSO (33 mM) in phosphate buffer (50 mM, pH 7.4), as well as the tested compounds (10 μL final concentration 100 μM) and ascorbic acid (10 mM) were mixed in test tubes and incubated at 37 °C for 30 min. The reaction was stopped by adding CCl_3_COOH (17% *w*/*v*), and the % competition activity of the nitrones **8** and **9** with DMSO for hydroxyl radicals was calculated. Trolox was used as a positive control.

#### 2.3.5. ABTS^∙+^–Decolorization Assay in Ethanolic Solution for Antioxidant Activity

ABTS stock solution in water (7 mM) was mixed with potassium persulfate (2.45 mM) and left in the dark at rt for 12–16 h before use, followed by the production of the ABTS radical cation (ABTS^+·^). The assay was performed as described previously [8]. The results were recorded after 1 min of the mixing solutions at 734 nm. Trolox was used as a positive control.

## 3. Results and Discussion

### 3.1. Chemistry

**Scheme 2:**
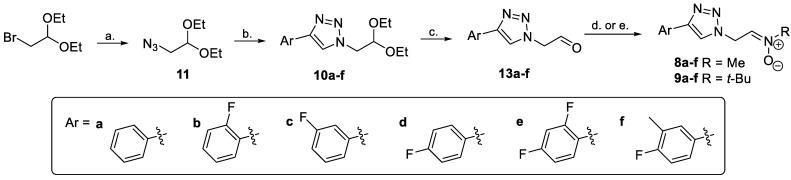
Synthesis of **8a**–**f** and **9a**–**f**. Reagents and conditions: a. NaN_3_, KI, DMSO, rt, and then 5 d at 90 °C; b. aryl alkyne **12a**–**f**, CuSO_4_ × 5H_2_O, sodium ascorbate, EtOH–H_2_O, 40–50 °C, 1 h, microwave (MW, 30 W); c. 1M HCl, 1 h, 120 °C; d. MeNHOH × HCl, CH_3_CO_2_Na, rt, 15 min; e. *t*-BuNHOH×HCl, CH_3_CO_2_Na, rt, 1 h.

The respective nitrones **8** and **9** were obtained, starting from commercially available 2-bromoacetaldehyde diethyl acetal, which was transformed into 2-azidoacetaldehyde diethyl acetal **11,** following the literature protocol [14]. Hüisgen dipolar cycloaddition of azide **11** with the selected aryl alkynes **12a**–**f** produced the respective 1,2,3-triazole cycloadducts **10a–f** in strong yields (80–94%), which were then efficiently hydrolyzed to the corresponding (1,2,3-triazole)aldehydes **13a**–**f** by treatment with 1M hydrochloric acid. The subsequent reactions with respective *N*-alkylhydroxylamine led to the formation of nitrones **8a**–**f** or **9a**–**f** in moderate-to-good yields (30–85%) (Scheme 2). While the full conversion of the aldehydes **13a**–**f** into *N*-methyl nitrones **8a**–**f** was achieved within 15 min at room temperature (rt), and the extension of the reaction time led to the formation of decomposition products, the synthesis of the *N*-*tert*-butyl nitrones **9a**–**f** required the stirring of aldehydes **13a**–**f** with *N*-*tert*-butylhydroxylamine for 1 h. All designed compounds were characterized by taking IR and ^1^H and ^13^C NMR spectra which, together with the correct elemental analyses, proved their structures (see Section 2.1. and Supplementary Material).

### 3.2. Antioxidant Activity of Nitrones **8a**–**f** and **9a**–**f**

In the present investigation, nitrones **8** and **9** were studied with regard to their antioxidant ability, as well as their ability to inhibit soybean LOX. In addition, standards nordihydroguaiaretic acid (NDGA), Trolox, and (*Z*)-*N-tert*-butyl-1-phenylmethanimine oxide (PBN) were included in the study for comparison. We decided to evaluate the in vitro antioxidant activity of the synthesized nitrones using three different antioxidant assays: (a) interaction with the water-soluble azo compound AAPH [2,2′-azobis(2-amidinopropane) dihydrochloride], (b) competition with the DMSO for hydroxyl radicals, and (c) ABTS^∙+^–decolorization assay in ethanolic solution for antioxidant activity, considering the role of ROS in OS and in inflammation disorders. Solubility or steric hindrance can vary among the different assays. Thus, the antioxidant ability of the compounds should be evaluated in a variety of media. The nitrones were also tested for their anti-inflammatory activity, as were lipoxygenase (LOX) inhibitors (LOXis).

The water-soluble azo compound AAPH has been extensively used as a clean and controllable source for the production of alkylperoxyl free radicals with the aid of temperature. The % inhibition of lipid peroxidation (ILP), using the APPH assay, by the examined compounds is shown in Table 1. Nitrones **8b**, **9c** and **9d** were found to be weak lipid peroxidation inhibitors (LPis) (2–29%). On the contrary, *t*-butyl nitrone **9e**, bearing the 2,4-difluorophenyl motif, showed a much higher inhibitory effect (100%), close to that of the reference compound Trolox (93%), being the most potent LPi in the whole series of tested nitrones. Furthermore, **8e** and **9f** present equipotent high ILP values (87%), followed by **9a** and **9b**, which are also equipotent. Lower antioxidant activities are recorded for **8c**, **8b** and **8f**.

Free hydroxyl radicals (^•^OH) are very harmful to the well-being of the human body, as they react with a number of biological important molecules such as DNA, lipids, or carbohydrates. Polyunsaturated fatty acids are found in high concentrations in the brain and are particularly vulnerable to free radicals. Thus, we found it of potential importance to test the ability of our nitrones to scavenge hydroxyl radicals. We used the competition of the synthesized nitrones with DMSO for HO^•^, generated by the Fe^3+^/ascorbic acid system and expressed as a percentage inhibition of formaldehyde production, to evaluate their hydroxyl radical scavenging activity. As shown in Table 1, the majority of the tested nitrones exhibited high activity at 100 µM. Among the representatives of group **8**, nitrones **8a** and **8f** compete strongly with DMSO for the hydroxyl radicals stronger than the standard compound Trolox. The scavenging activity for nitrones **8b**, **8c**, **8d**, **8e** ranged from 59–80%. For nitrones of group **9**, the scavenging activities were found to be higher than that of group **8**, especially for **9c**, **9d**, **9e** and **9f** (89–100%). In addition, they were higher than those of Trolox (88%), used as a reference compound. Nitrones **9d**, **9e** and **9f**, bearing the 4-fluorophenyl, 2,4-difluorophenyl, and 4-fluoro-3-methylphenyl motifs, respectively, were almost equipotent and represented the most potent hydroxyl radical scavengers (~100%). We noticed that the presence of fluorine as a substituent in the phenyl ring, as well as its specific position, respectively, influences the scavenging activity, and that lipophilicity is not correlated with these results.

LOX is one of the enzymes implicated in the first two steps in the metabolism of arachidonic acid to leukotrienes. The generation of LTB4 is important in the pathogenesis of neutrophil-mediated inflammatory diseases related to the severity of cardiovascular diseases, asthma, and cancer. Published researches suggest the relationship between LOX inhibition and the ability of the inhibitors to reduce Fe^3+^ at the active site to the catalytically inactive Fe^2+^. However, alternative mechanisms suggest that most of the LOXis are antioxidants or free radical scavengers. A perusal of the IC_50′_s inhibition values (Table 1) showed that between the two groups **8** and **9**, the *t*-butyl analogues were found to be more potent. Thus, the most potent inhibitor was the *t*-butyl substituted nitrone **9f** (27 μM), bearing the 4-fluoro-3-methylphenyl motif, 60-fold less potent than NDGA (0.45 μM), used as standard in this test, followed by **9b**, **9d** ⋍ **9e**. Among the members of group **8**, the most potent **8a** presents a low log *p* value and is followed by **8e**. We noticed that the presence of fluorine as a substituent in the phenyl ring, as well as its specific position, respectively, influences the inhibitory activity. The IC_50_ value of the simplest nitrone **8a** is 37.5 μM, whereas the 4-fluorophenyl nitrone (**8d**) loses its activity. The observed activity is very low when fluorine is inserted at the 2- or 3^-^position of phenyl residue (24 and 40% for **8b** and **8c**, respectively), as well as when a methyl group is inserted next to the fluorine substituent (44%) (**8f**). Fluorine substitution at both 2- and 4-positions of phenyl group lowers the inhibition (**8e**) in comparison to **8a**. Although lipophilicity is referred to as an important physicochemical property for LOXis, herein the theoretically calculated log *P* values do not always support this observation. Of course, the most potent LOXi **9f** presents the highest log *P* value (2.12) within both groups. The presence of fluorine as a substituent on the phenyl ring, as well as its specific position, respectively, influences the inhibitory activity. Thus, the substitution with fluorine at the 2-position of phenyl group gives an IC_50_ response of 40 μM (**9b**), whereas the substation at 3-position (**9c**) is related to no inhibition under the reported experimental conditions. The 4-fluorophenyl nitrone points to the inhibition of 100µM (**9d**). The 2,4-diflurophenyl nitrone (**9e**) is equipotent to the **9d**. The replacement of the methyl group by hydrogen diminishes inhibition (**9d)**.

The results from the antioxidant determination in the ABTS radical cation (ABTS^•+^) decolorization assay were not significant. *N*-Methyl nitrone **8f**, bearing the 4-fluoro-3-methylphenyl motif, was the only promising representative with a value of 34.3%, followed by nitrone **9f** (16%), whereas **8a**, **8b**, **8d**, and **9a** showed very limited antioxidant activity (4–14%).

## 4. Conclusions

In this work, we have described the design, synthesis, and antioxidant capacity of six novel *N*-(2-(4-aryl-1*H*-1,2,3-triazol-1-yl)ethylidene)methanamine oxides **8a**–**f** and six novel *N*-(2-(4-aryl)-1*H*-1,2,3-triazol-1-yl)ethylidene)-2-methylpropan-2-amine oxides **9a**–**f**, bearing an *N*-methyl, and an *N-t*-butyl, respectively, at the nitrogen of the nitrone motif (Scheme 1).

Based on the hypothesis that increasing the potency of an antioxidant agent may be achieved by combining two or even more structural and functional motifs with proven antioxidant activity, the triazole heterocyclic ring system has been selected for designing new nitrones, since several examples of antioxidants bearing the triazole core are known (Figure 3). Consequently, nitrones **8a**–**f** and **9a**–**f** were prepared by simple methods and in a short synthetic scheme from commercial and easily available precursors (Scheme 2).

Next, nitrones **8** and **9** have been investigated for their antioxidant ability, as well as for their capacity to inhibit soybean LOX, and the in vitro antioxidant activity. This was carried out using three different antioxidant assays, such as the interaction with the water-soluble azo compound AAPH for the ILP test, the competition with the DMSO for hydroxyl radicals, and the ABTS^•+^–decolorization assay.

*t*-Butyl nitrone **9e**, bearing the 2,4-difluorophenyl motif, showed a strong inhibitory effect (100%), close to the reference compound Trolox (93%), being the most potent LPi of the whole series of tested nitrones. Nitrones **9d**, **9e** and **9f**, bearing the 4-fluorophenyl, 2,4-difluorophenyl, and 4-fluoro-3-methylphenyl motifs, respectively, were almost equipotent, and constituted the most potent hydroxyl radical scavengers (~100%), more potent than Trolox (88%), used as a reference compound. Regarding the LOX inhibition, the most potent inhibitor was the *t*-butyl-substituted nitrone **9f** (27 μM), bearing the 4-fluoro-3-methylphenyl motif, being 60-fold less potent than NDGA (0.45 μM), used as the standard in this test. The results from the antioxidant determination in the ABTS radical cation (ABTS^•+^) decolorization assay were not significant. *N*-Methyl nitrone **8f**, bearing the 4-fluoro-3-methylphenyl motif, was the only promising representative, with a value of 34.3%, followed by nitrone **9f** (16%). Conversely, **8a**, **8b**, **8d**, and **9a** showed very limited antioxidant activity (4–14%).

Overall, we have identified *N*-(2-(4-(4-fluoro-3-methylphenyl)-1*H*-1,2,3-triazol-1-yl)ethylidene)-2-methylpropan-2-amine oxide (**9f**), bearing *t*-butyl and 4-fluoro-3-methylphenyl motifs in its structure, as the most balanced and potent antioxidant agent among the tested nitrones. As shown in Table 1, nitrone **9f** was the most potent LOXi (27 μM), an extremely efficient and potent hydroxyl radical scavenger (99.9%), and one of the most potent LPis (87%) and ABTS^•+^ scavengers (16%) of the whole series of tested nitrones. Finally, nitrone **9f** compared satisfactorily with standards Trolox, and NDGA, and particularly very well with standard PBN, as shown in Table 1.

To sum up, we think that compound **9f** is a very promising hit nitrone that, based on the present results, deserves further investigation on biological targets involved in pathologies, such as stroke or Alzheimer’s disease, where OS is at the origin of their progress and development.

## Figures and Tables

**Table 1:** In vitro antioxidant activities of nitrones **8a**–**f** and **9a**–**f**.

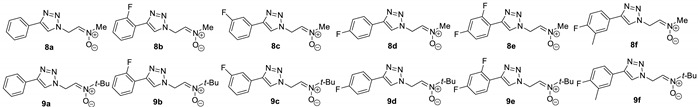					
Nitrones/ Standards	Clog *P* ^b^	LOX Inhibition (% ^a^ or IC_50_ [µM])	ILP (%) ^a^	Scavenging Activity for HO^•^ (%) ^a^	ABTS^+∙^ (%) ^a^
**8a**	0.21	37.5 μM	48.4	93	14
**8b**	0.39	24%	2	61	13
**8c**	0.39	40%	65	59	no
**8d**	0.39	no	54	80	5.3
**8e**	0.54	87.5 μM	**87**	76	no
**8f**	0.59	44 %	41	91.5	**34.3**
**9a**	1.44	44%	74.5	76	4
**9b**	1.62	40 μM	73	76	no
**9c**	1.44	no	21	89	no
**9d**	1.62	100 μM	29	**99**	no
**9e**	1.42	100 μM	**100**	**100**	no
**9f**	2.12	**27 μM**	**87**	**99.9**	**16**
NDGA		87 (0.45 μM)			
Trolox			93	88	91
PBN		23%	11	no	5

^a^ Nitrones tested at 100 µM. Values are means of three or four different determinations. no = no activity under the experimental conditions. Means within each column differ significantly (*p* < 0.05). ^b^ Biobyte BioByte Corporation, C-QSAR database, 201 W Fourth Str., Suite # 204, Claremont, CA 91711-4707, USA.

## Data Availability

Data is contained within the article and supplementary material.

## Supplementary Materials

The following supporting information can be downloaded at: https://www.mdpi.com/article/10.3390/antiox12010036/s1, Figure S1: ^1^H NMR Spectrum for **8a** in CDCl_3_; Figure S2: ^13^C NMR Spectrum for **8a** in CDCl_3_; Figure S3: ^1^H NMR Spectrum for **8b** in CDCl_3_; Figure S4: ^13^C NMR Spectrum for **8b** in CDCl_3_; Figure S5: ^1^H NMR Spectrum for **8c** in D_2_O; Figure S6: ^13^C NMR Spectrum for **8c** in CDCl_3_; Figure S7: ^1^H NMR Spectrum for **8d** in D_2_O; Figure S8: ^13^C NMR Spectrum for **8d** in CDCl_3_; Figure S9: ^1^H NMR Spectrum for **8e** in D_2_O; Figure S10: ^13^C NMR Spectrum for **8e** in CDCl_3_; Figure S11: ^1^H NMR Spectrum for **8f** in D_2_O; Figure S12: ^13^C NMR Spectrum for **8f** in CDCl_3_; Figure S13: ^1^H NMR Spectrum for **9a** in CDCl_3_; Figure S14: ^13^C NMR Spectrum for **9a** in CDCl_3_; Figure S15: ^1^H NMR Spectrum for **9b** in CDCl_3_; Figure S16: ^13^C NMR Spectrum for **9b** in CDCl_3_; Figure S17: ^1^H NMR Spectrum for **9c** in CDCl_3_; Figure S18: ^13^C NMR Spectrum for **9c** in CDCl_3_; Figure S19: ^1^H NMR Spectrum for **9d** in CDCl_3_; Figure S20: ^13^C NMR Spectrum for **9d** in CDCl_3_; Figure S21: ^1^H NMR Spectrum for **9e** in CDCl_3_; Figure S22: ^13^C NMR Spectrum for **9e** in CDCl_3_; Figure S23: ^1^H NMR Spectrum for **9f** in D_2_O; Figure S24: ^13^C NMR Spectrum for **9f** in CDCl_3_.

## Abbreviations

AAPH, 2,2′-Azobis(2-amidinopropane) dihydrochloride; ILP, Inhibition of lipid peroxydation; LOX, Lipoxygenase; LP, Lipid peroxidation; NDGA, Nordihydroguaretic acid; OS, Oxidative stress; PNB, α-phenyl-N-tert-butylnitrone; ROS, Reactive Oxygen Species; RNS, Reactive Nitrogen Species; NXY-059, Disodium 2,4-sulphophenyl-*N*-tert-butylnitrone; QN23, (*Z*)-*N*-*tert*-butyl-1-(2-chloro-6-methoxyquinolin-3-yl)methanimine oxide; 4-OHPBN, α-4-hydroxyphenyl-*N*-tert-butylnitrone.
