# Supplementary material for: Synthesis and Antioxidant Properties of Novel 1,2,3-Triazole-Containing Nitrones
Source: Antioxidants (Basel). 2022 Dec 24;12(1):36. doi: 10.3390/antiox12010036 (PMC9854728; doi:10.3390/antiox12010036)
Supplement: Supplementary file 1 [file antioxidants-12-00036-s001.zip › antioxidants-2120168-supplementary.pdf]

Synthesis and antioxidant properties of novel 1,2,3-triazole-containing nitrones

Dimitra Hadjipavlou-Litina [1,*], Iwona E. Głowacka[2], Jose Marco-Contelles[3,4], Dorota G. Piotrowska [2,*]

1 Department of Pharmaceutical Chemistry, School of Pharmacy, Faculty of Health Sciences,

Aristotle University of Thessaloniki, 54124 Thessaloniki, Greece

2 Bioorganic Chemistry Laboratory, Faculty of Pharmacy, Medical University of Lodz, Muszy´ nskiego 1,

90-151 Lodz, Poland

3 Laboratory of Medicinal Chemistry, Institute of Organic Chemistry (CSIC), Juan de la Cierva 3,

28006 Madrid, Spain

4 Centre for Biomedical Network Research on Rare Diseases (CIBERER), CIBER, ISCIII, 46010 Madrid, Spain

* Correspondence: hadjipav@pharm.auth.gr (D.H.-L.); dorota.piotrowska@umed.lodz.pl (D.G.P.);

Tel.: +30-23-1099-7627 (D.H.-L.); +48-42-677-92-33 (D.G.P.)

Contents

NMR spectra for compounds **8a-8f** and **9a-9f**:

Figure S1: ^{1}H NMR Spectrum for **8a** in $CDCl_3$

Figure S2: ^{13}C NMR Spectrum for **8a** in $CDCl_3$

Figure S3: ^{1}H NMR Spectrum for **8b** in $CDCl_3$

Figure S4: ^{13}C NMR Spectrum for **8b** in $CDCl_3$

Figure S5: ^{1}H NMR Spectrum for **8c** in D_2O

Figure S6: ^{13}C NMR Spectrum for **8c** in $CDCl_3$

Figure S7: ^{1}H NMR Spectrum for **8d** in D_2O

Figure S8: ^{13}C NMR Spectrum for **8d** in $CDCl_3$

Figure S9: ^{1}H NMR Spectrum for **8e** in D_2O

Figure S10: ^{13}C NMR Spectrum for **8e** in $CDCl_3$

Figure S11: ^{1}H NMR Spectrum for **8f** in D_2O

Figure S12: ^{13}C NMR Spectrum for **8f** in $CDCl_3$

Figure S13: ^{1}H NMR Spectrum for **9a** in $CDCl_3$

Figure S14: ^{13}C NMR Spectrum for **9a** in $CDCl_3$

Figure S15: ^{1}H NMR Spectrum for **9b** in $CDCl_3$

Figure S16: ^{13}C NMR Spectrum for **9b** in $CDCl_3$

Figure S17: ^{1}H NMR Spectrum for **9c** in $CDCl_3$

Figure S18: ^{13}C NMR Spectrum for **9c** in $CDCl_3$

Figure S19: ^{1}H NMR Spectrum for **9d** in $CDCl_3$

Figure S20: ^{13}C NMR Spectrum for **9d** in $CDCl_3$

Figure S21: ^{1}H NMR Spectrum for **9e** in $CDCl_3$

Figure S22: ^{13}C NMR Spectrum for **9e** in $CDCl_3$

Figure S23: ^{1}H NMR Spectrum for **9f** in D_2O

Figure S24: ^{13}C NMR Spectrum for **9f** in $CDCl_3$

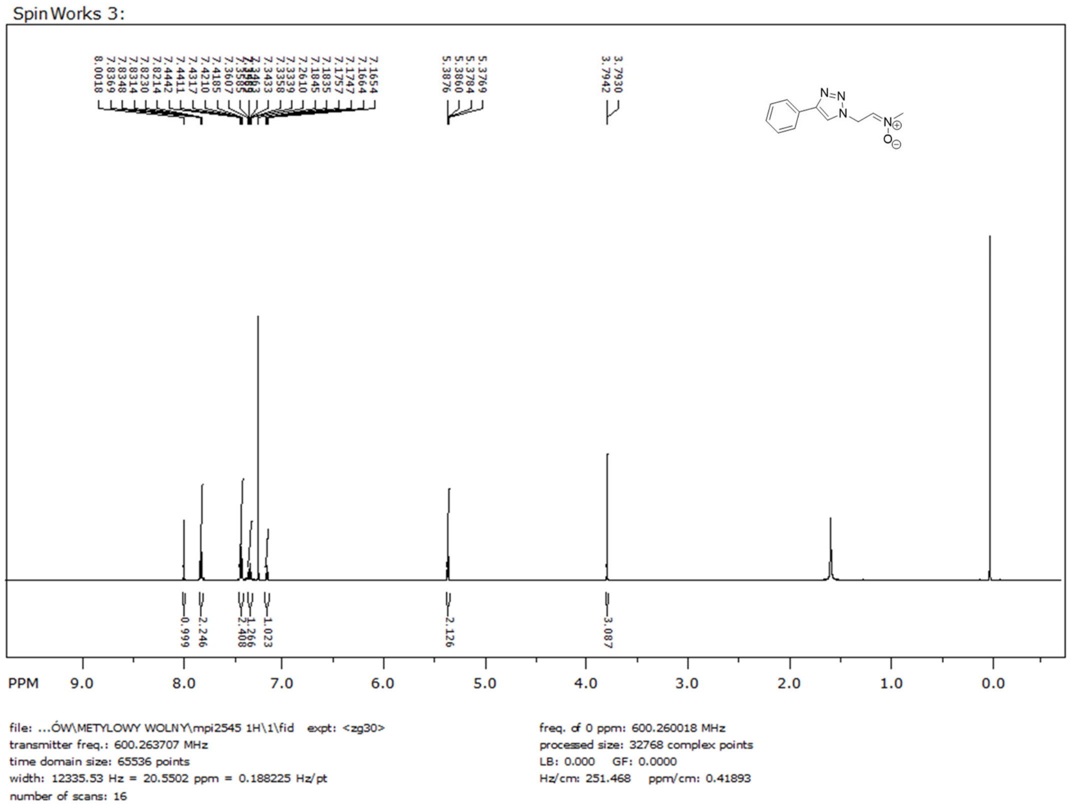

Figure S1: ^{1}H NMR Spectrum for **8a** in $CDCl_3$

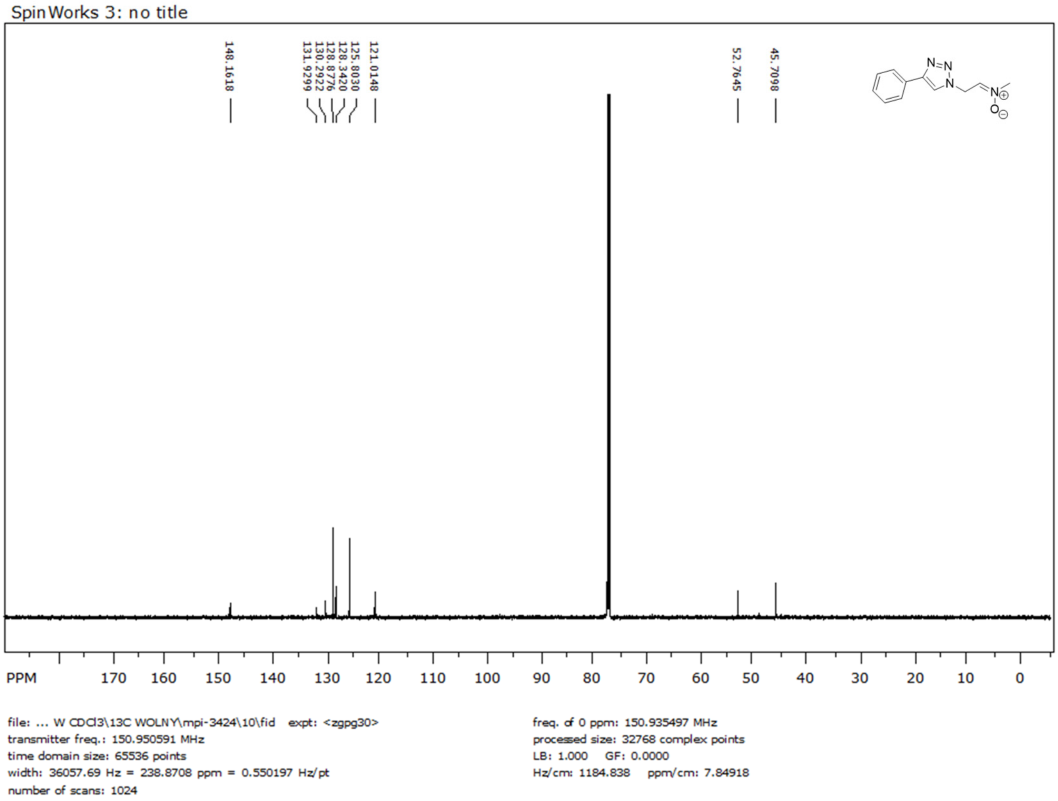

Figure S2: ^{13}C NMR Spectrum for **8a** in $CDCl_3$

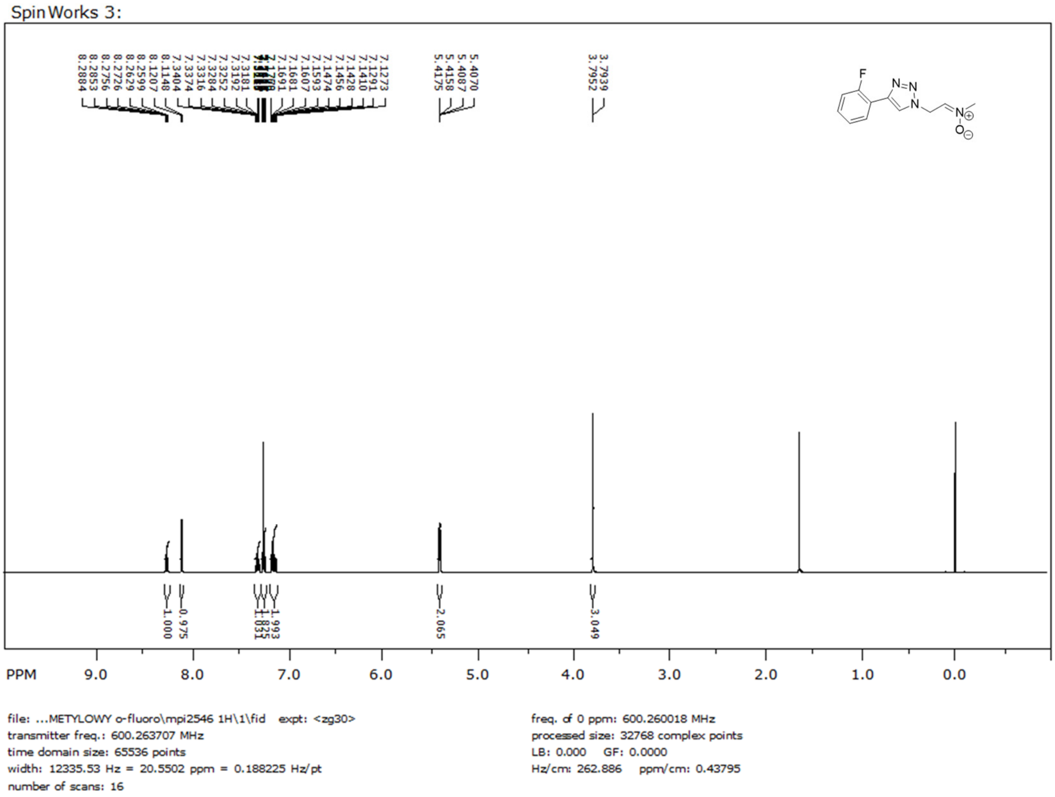

Figure S3: ^{1}H NMR Spectrum for **8b** in $CDCl_3$

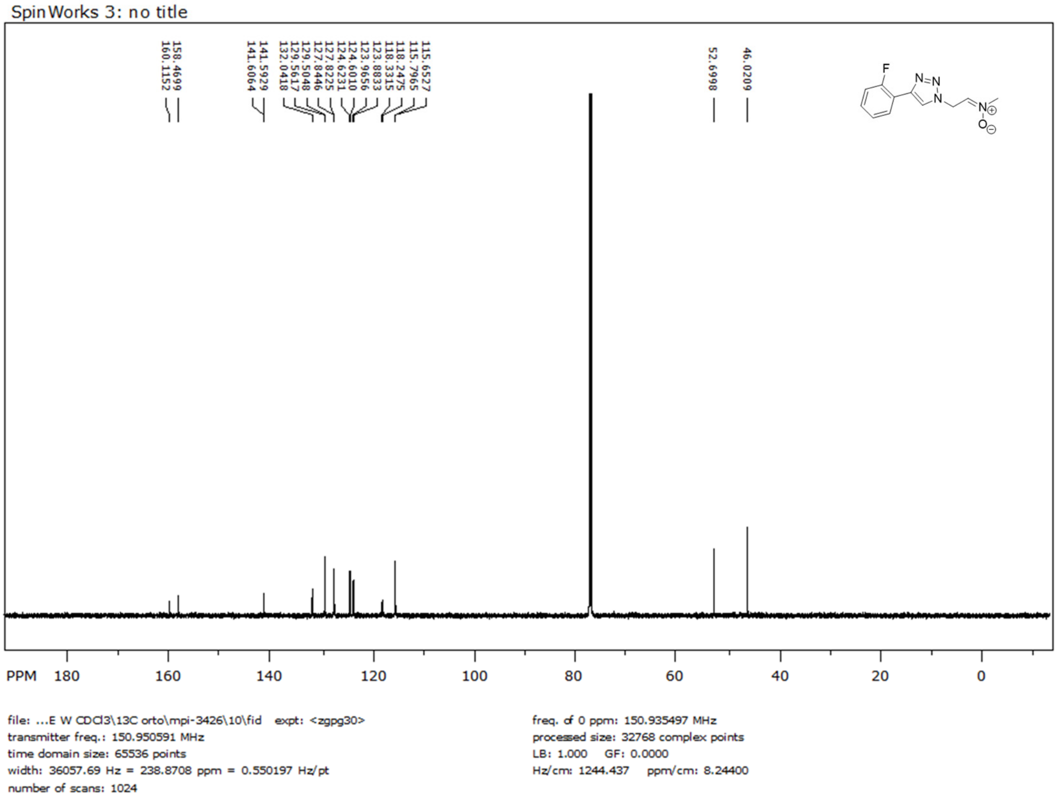

Figure S4: ^{13}C NMR Spectrum for **8b** in $CDCl_3$

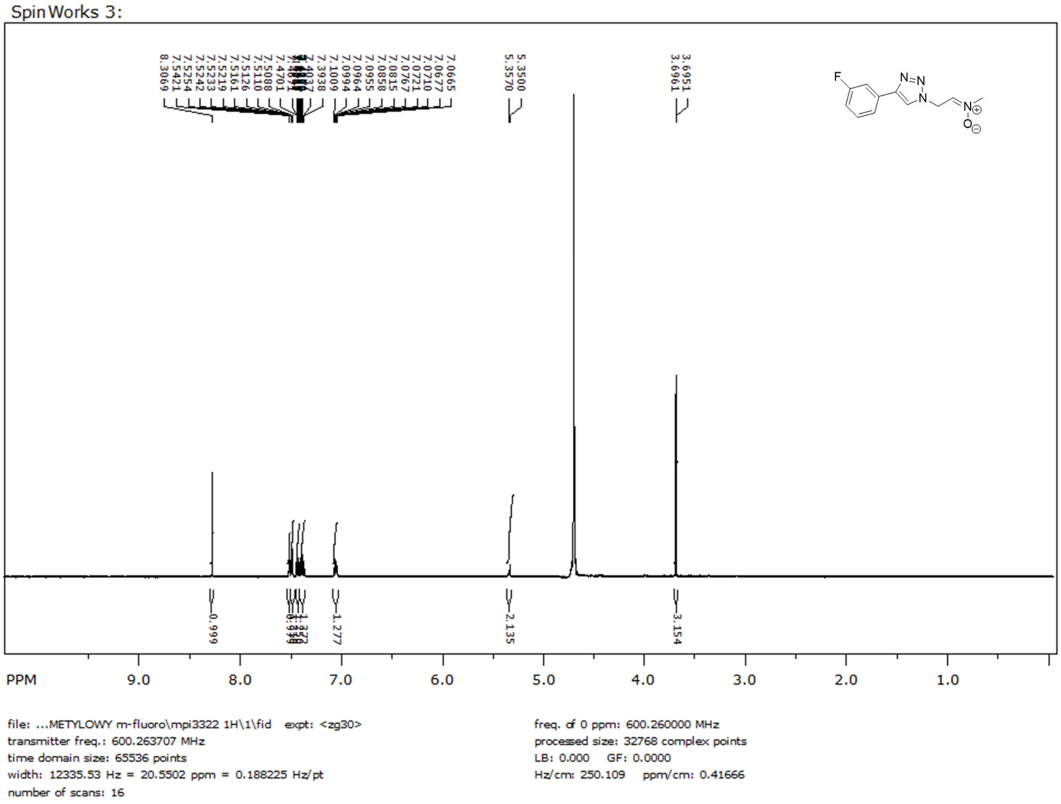

Figure S5: ^{1}H NMR Spectrum for **8c** in D_2O

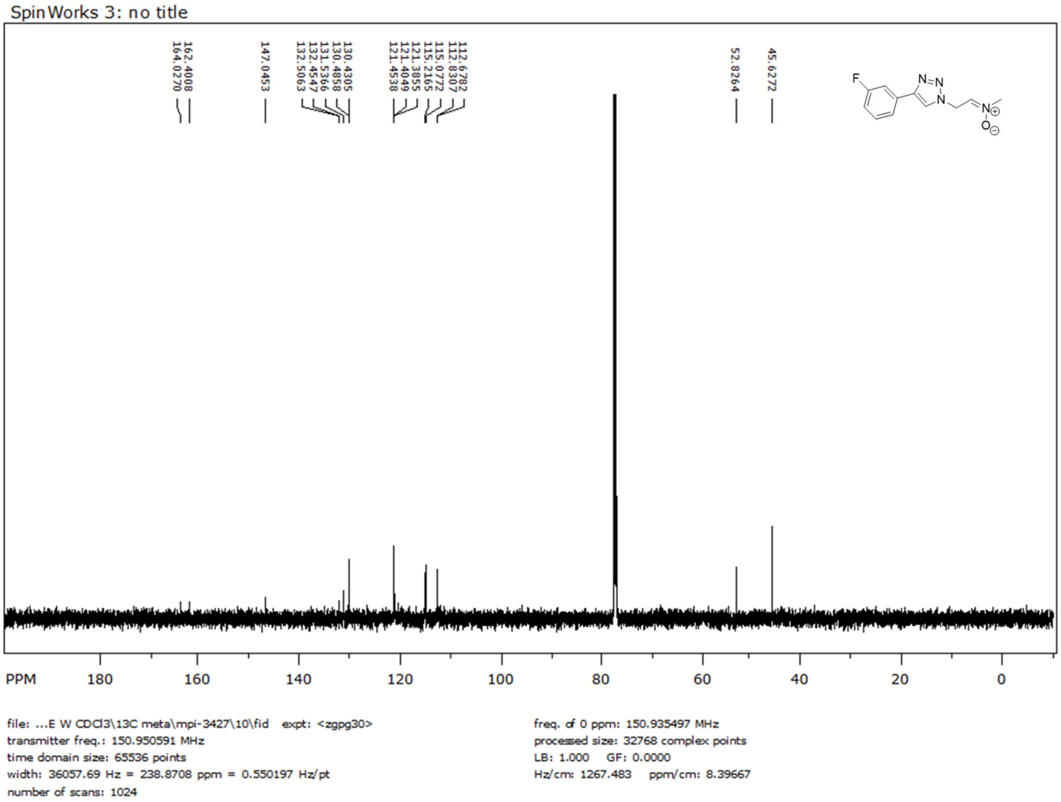

Figure S6: ^{13}C NMR Spectrum for **8c** in $CDCl_3$

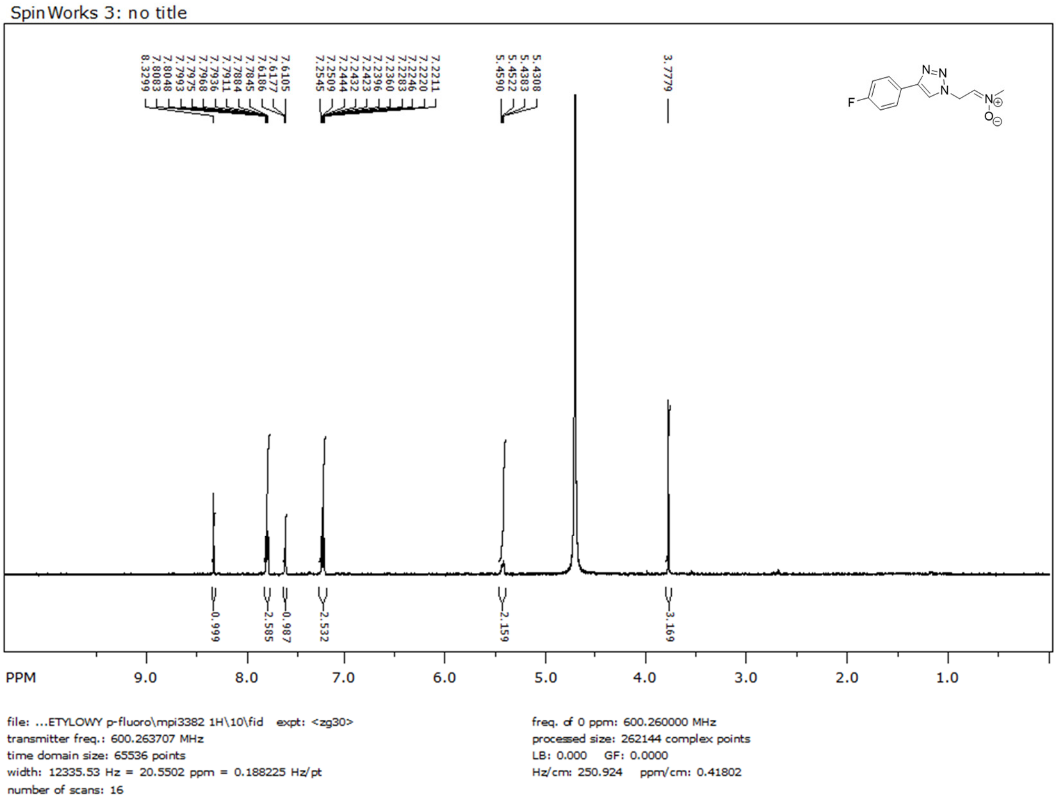

Figure S7: ^{1}H NMR Spectrum for **8d** in D_2O

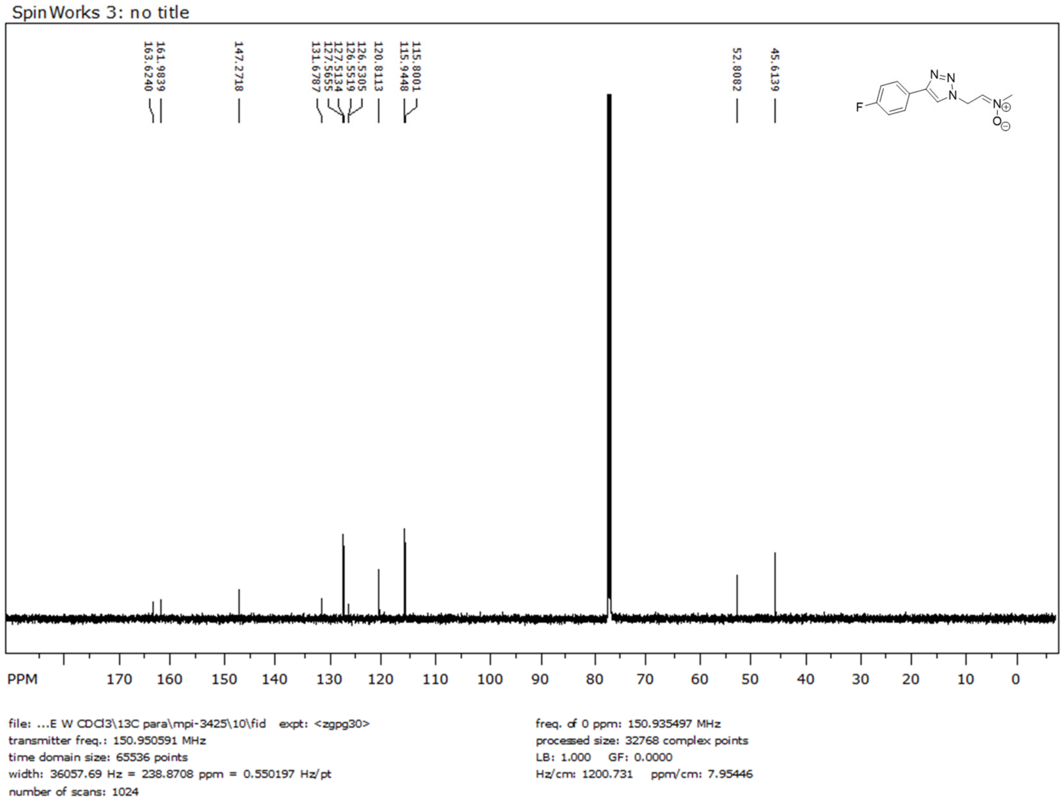

Figure S8: ^{13}C NMR Spectrum for **8d** in $CDCl_3$

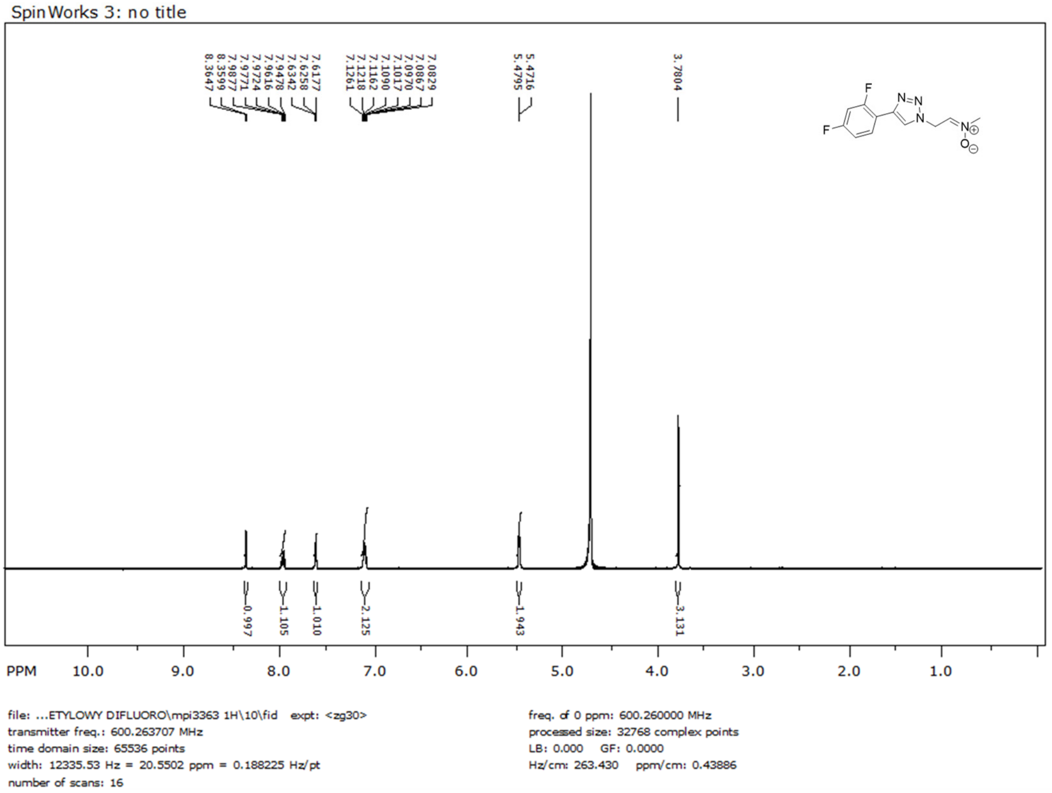

Figure S9: ^{1}H NMR Spectrum for **8e** in D_2O

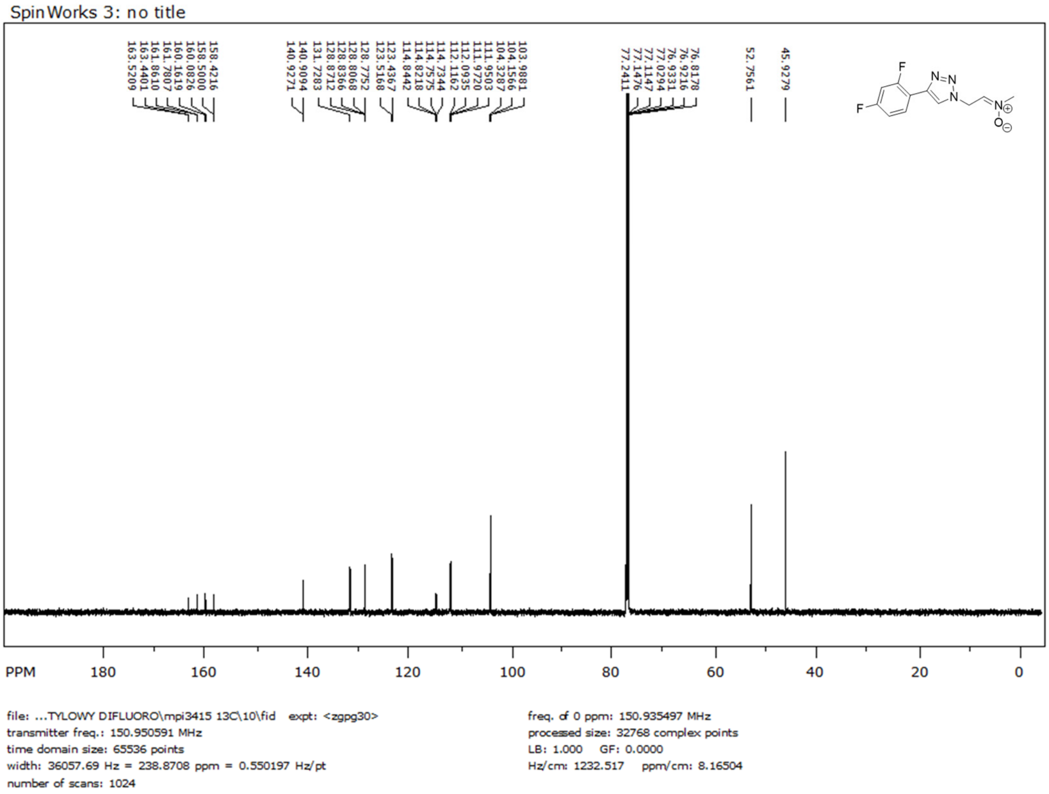

Figure S10: ^{13}C NMR Spectrum for **8e** in $CDCl_3$

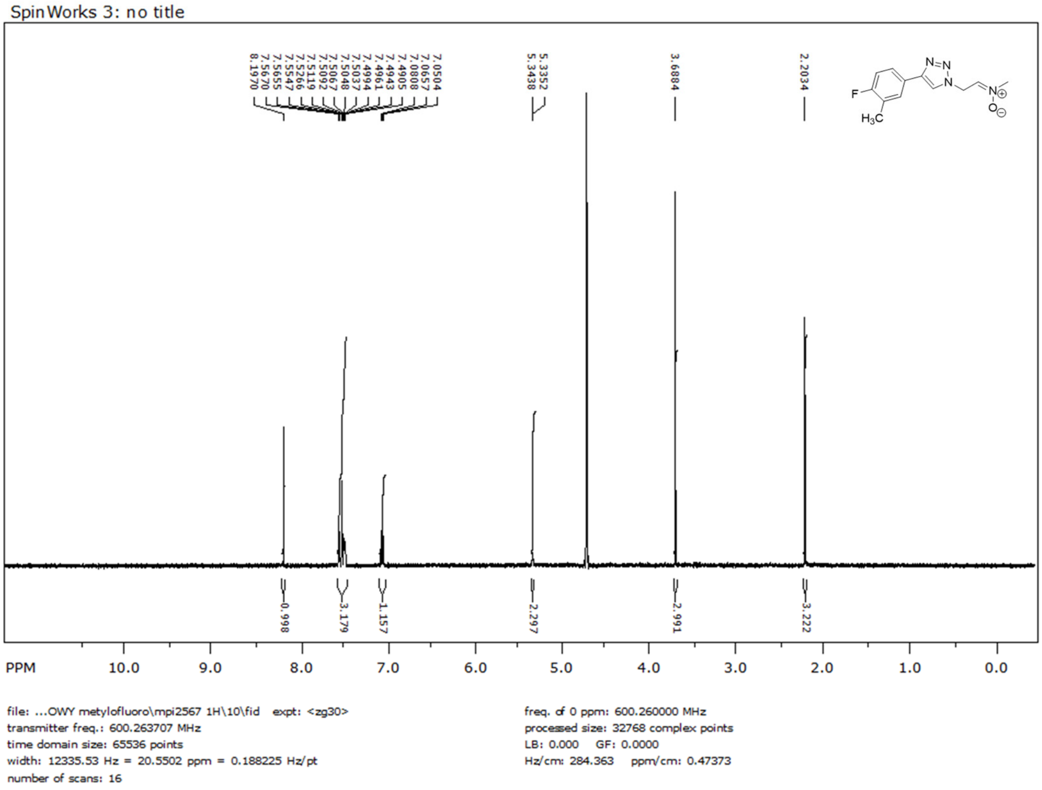

Figure S11: ^{1}H NMR Spectrum for **8f** in D_2O

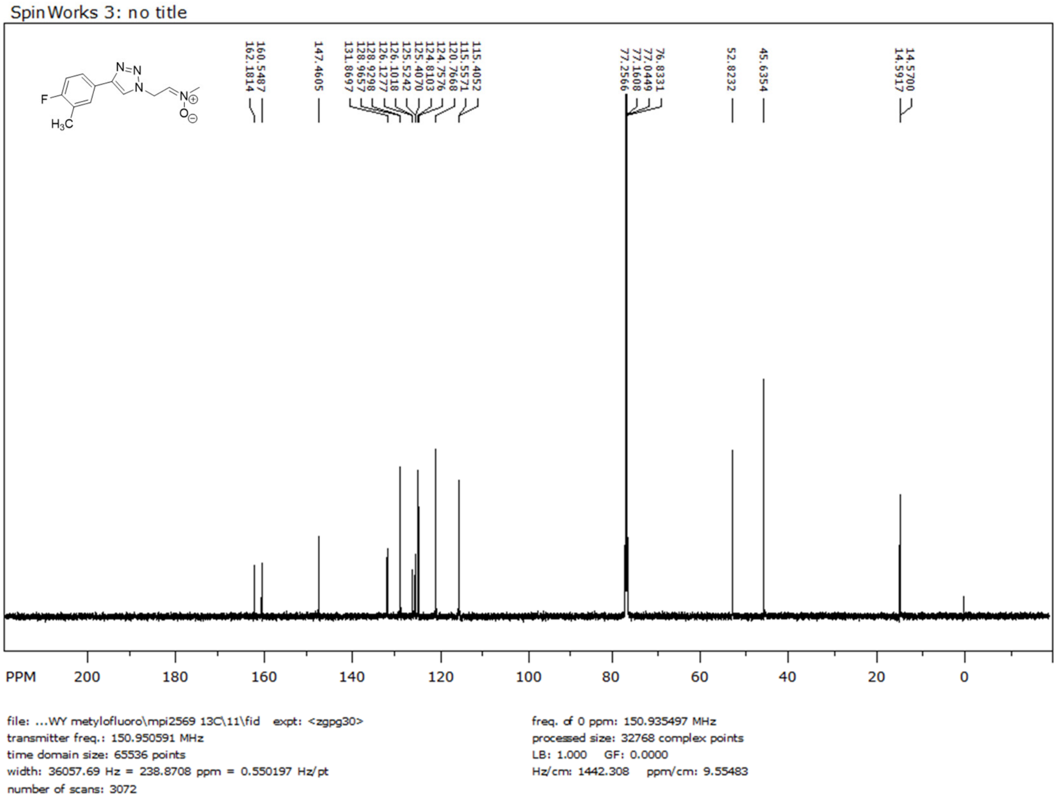

Figure S12: ^{13}C NMR Spectrum for **8f** in $CDCl_3$

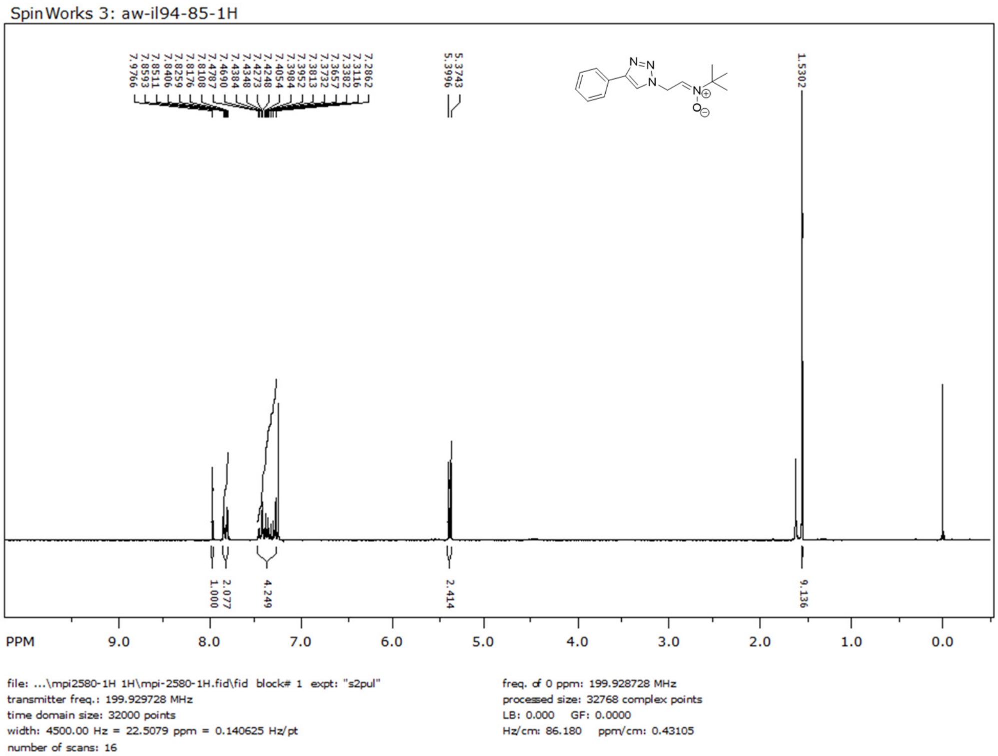

Figure S13: ^{1}H NMR Spectrum for **9a** in $CDCl_3$

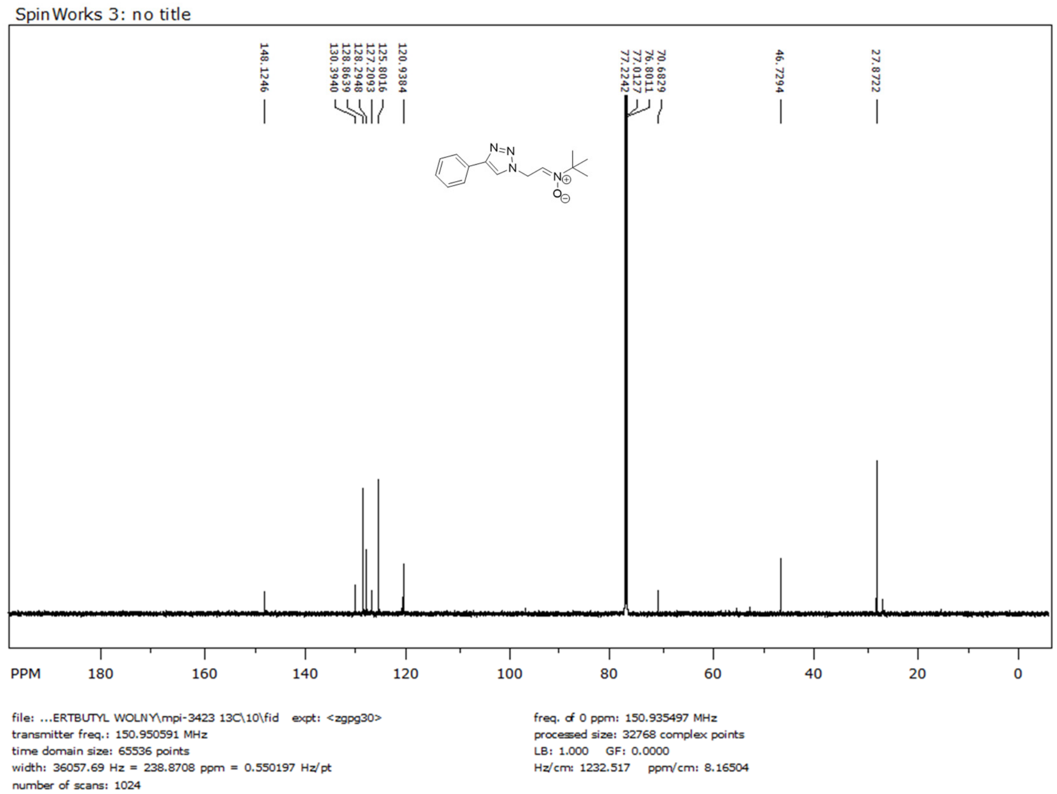

Figure S14: ^{13}C NMR Spectrum for **9a** in $CDCl_3$

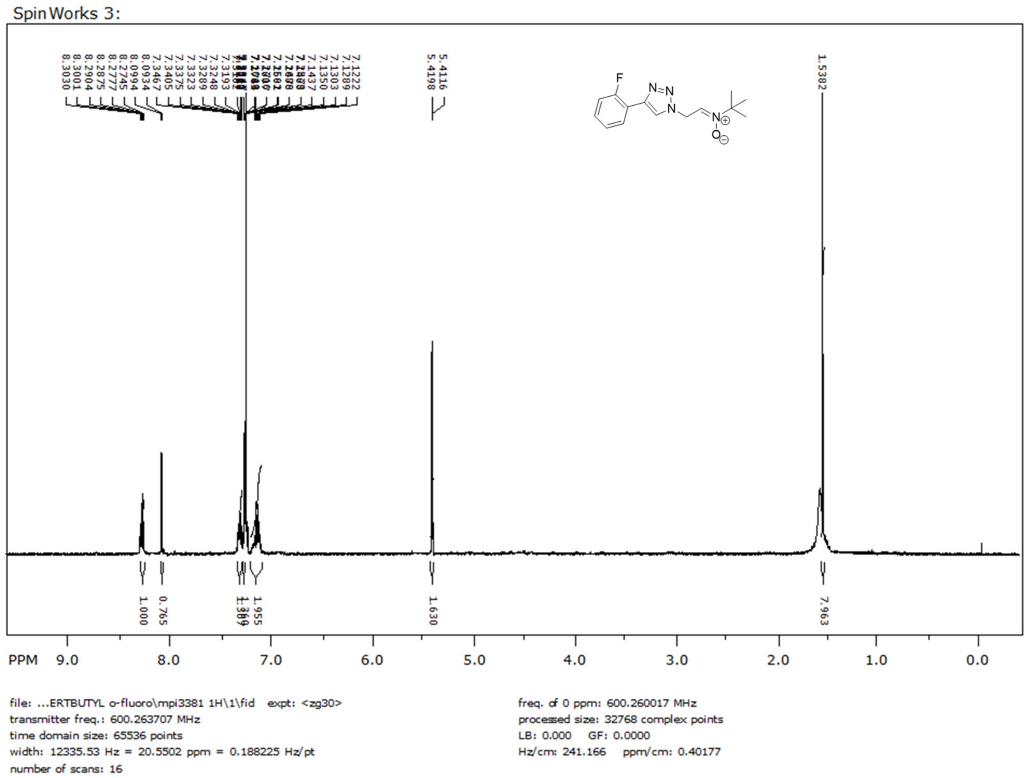

Figure S15: ^{1}H NMR Spectrum for **9b** in $CDCl_3$

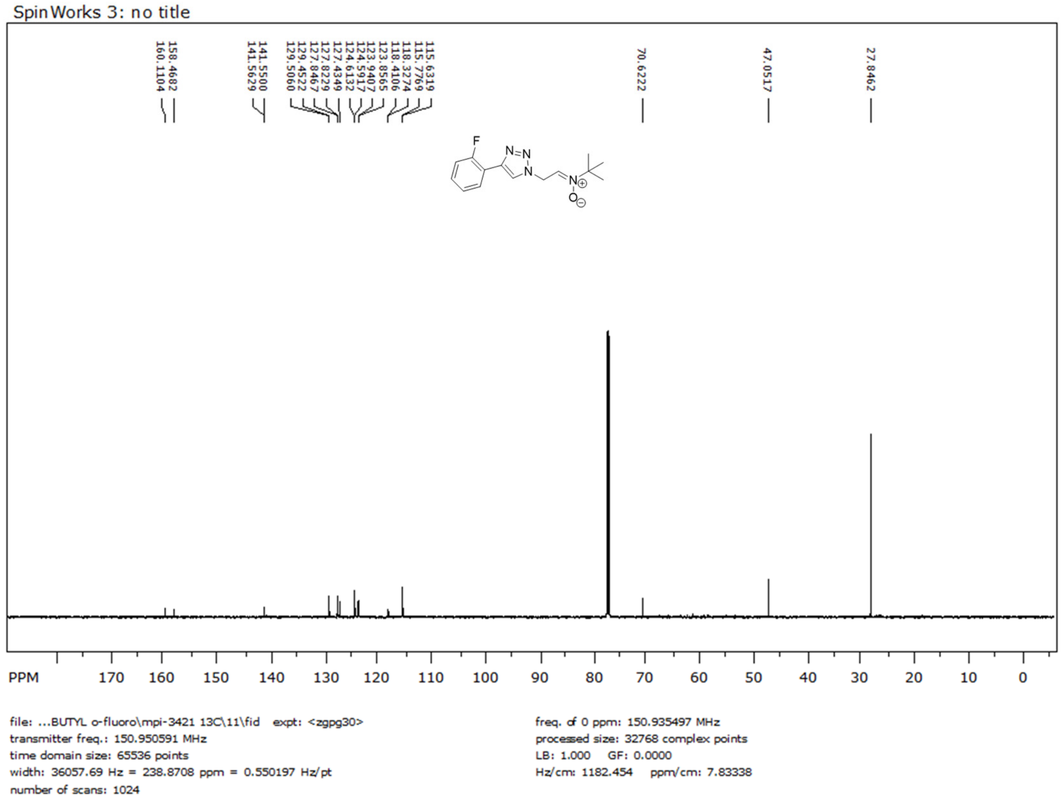

Figure S16: ^{13}C NMR Spectrum for **9b** in $CDCl_3$

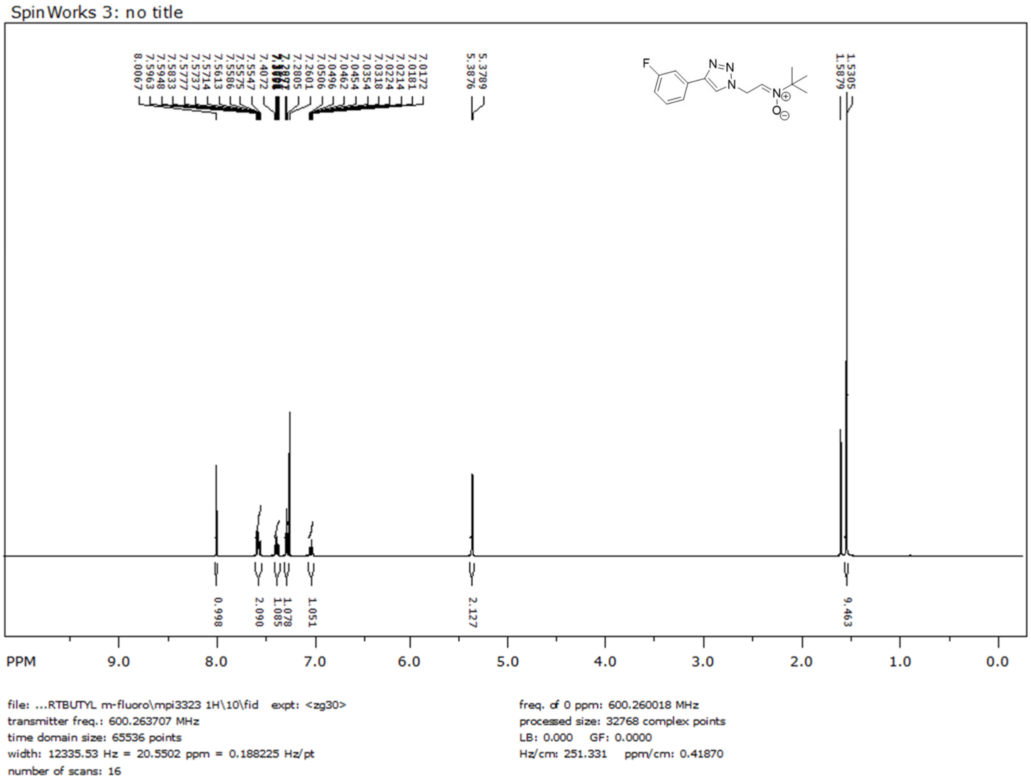

Figure S17: ^{1}H NMR Spectrum for **9c** in $CDCl_3$

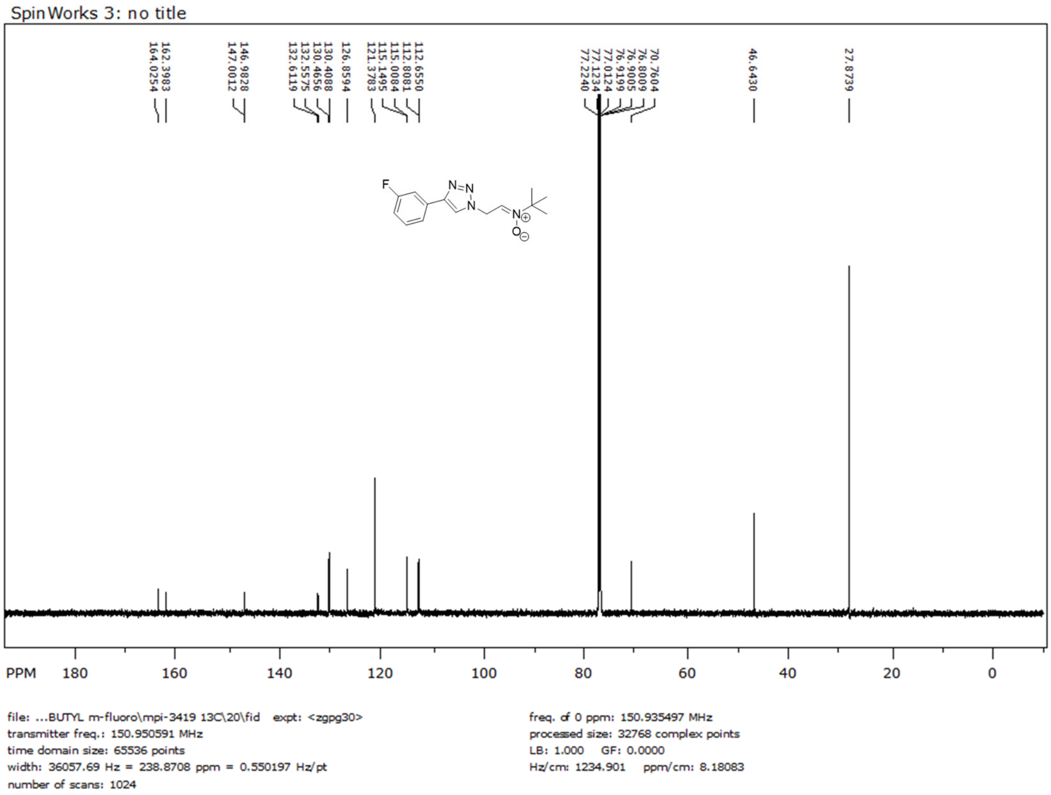

Figure S18: ^{13}C NMR Spectrum for **9c** in $CDCl_3$

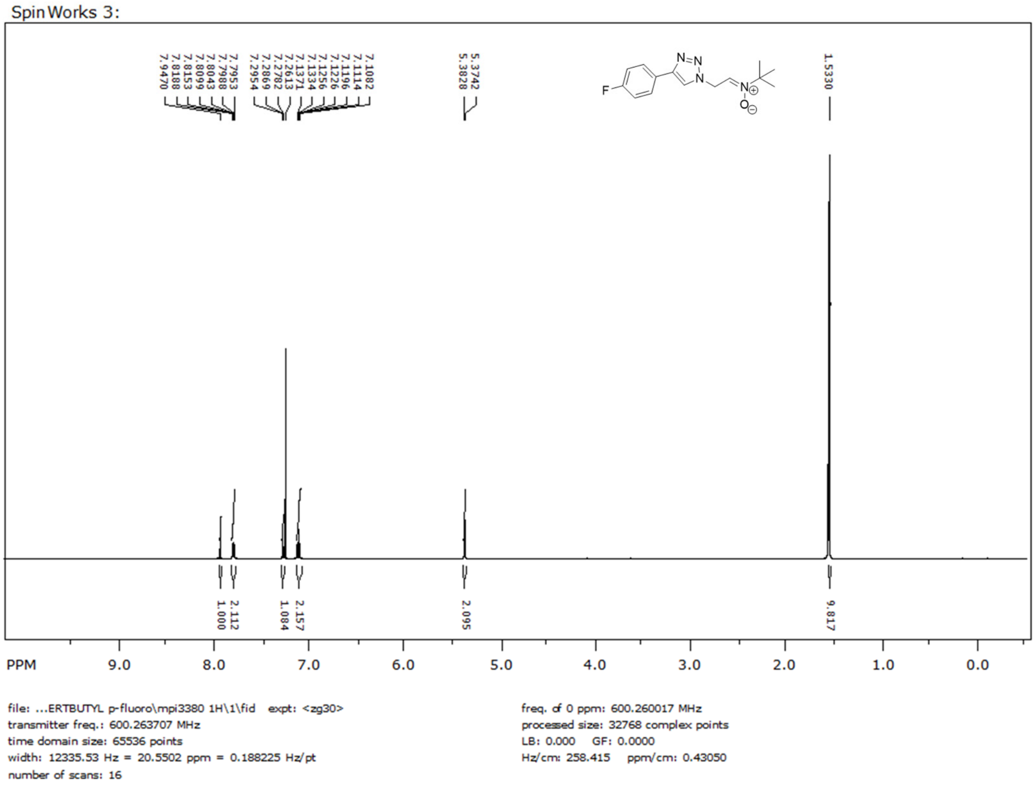

Figure S19: ^{1}H NMR Spectrum for **9d** in $CDCl_3$

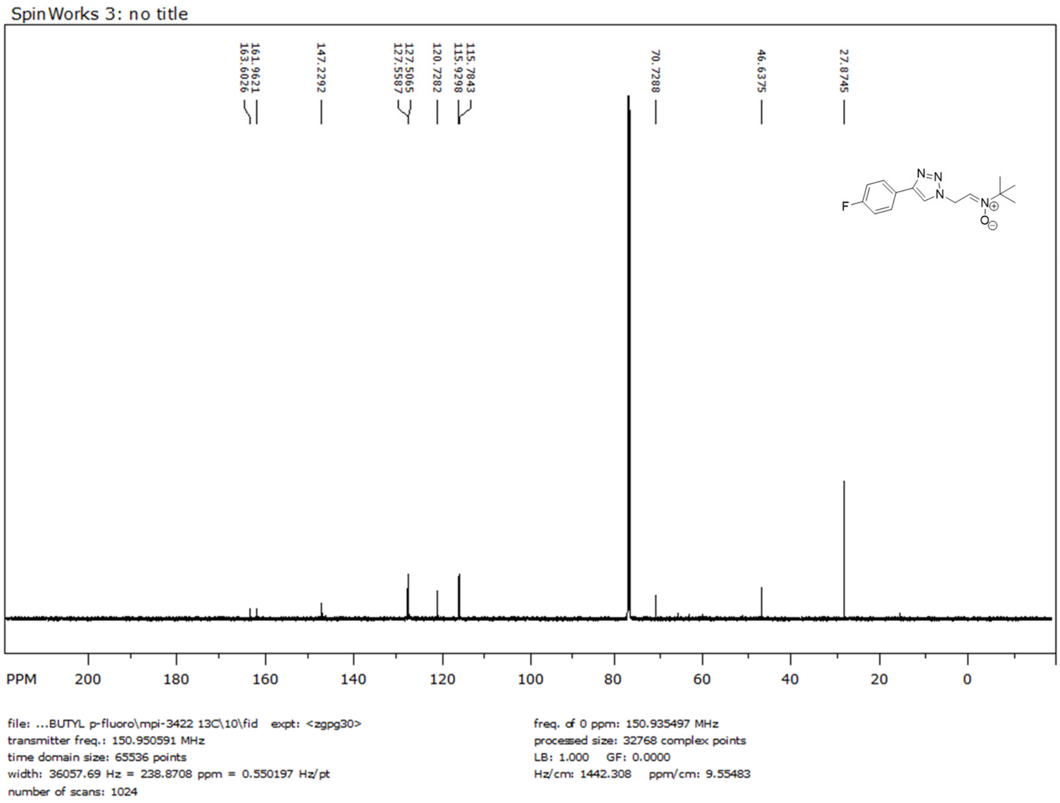

Figure S20: ^{13}C NMR Spectrum for **9d** in $CDCl_3$

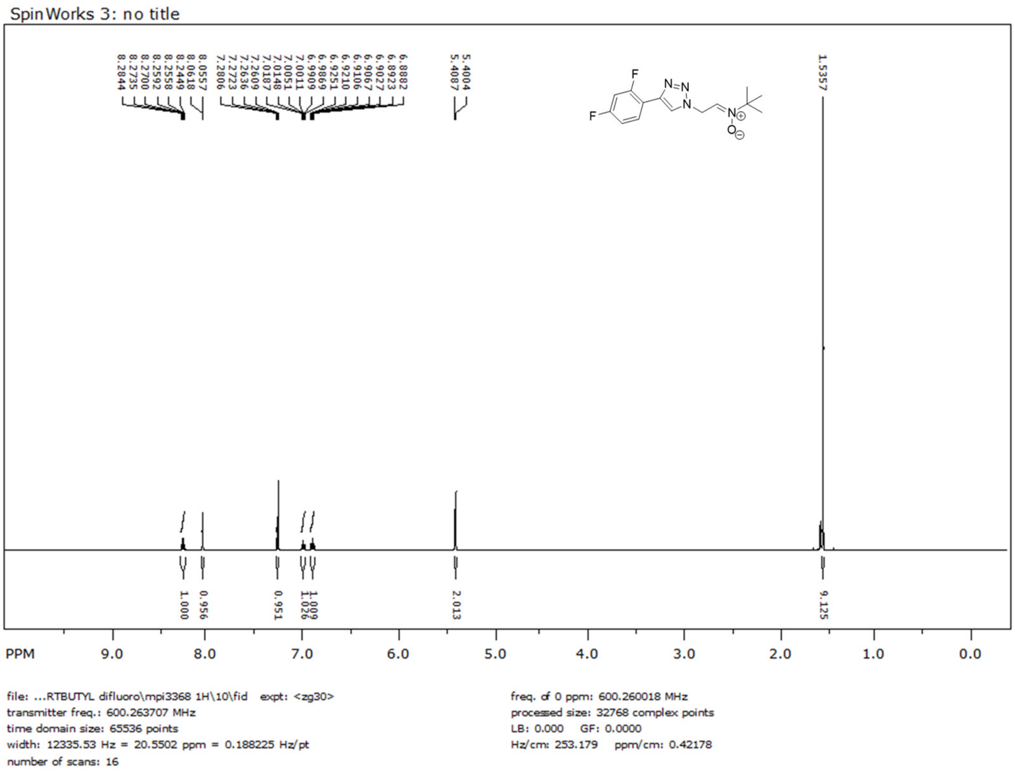

Figure S21: ^{1}H NMR Spectrum for **9e** in $CDCl_3$

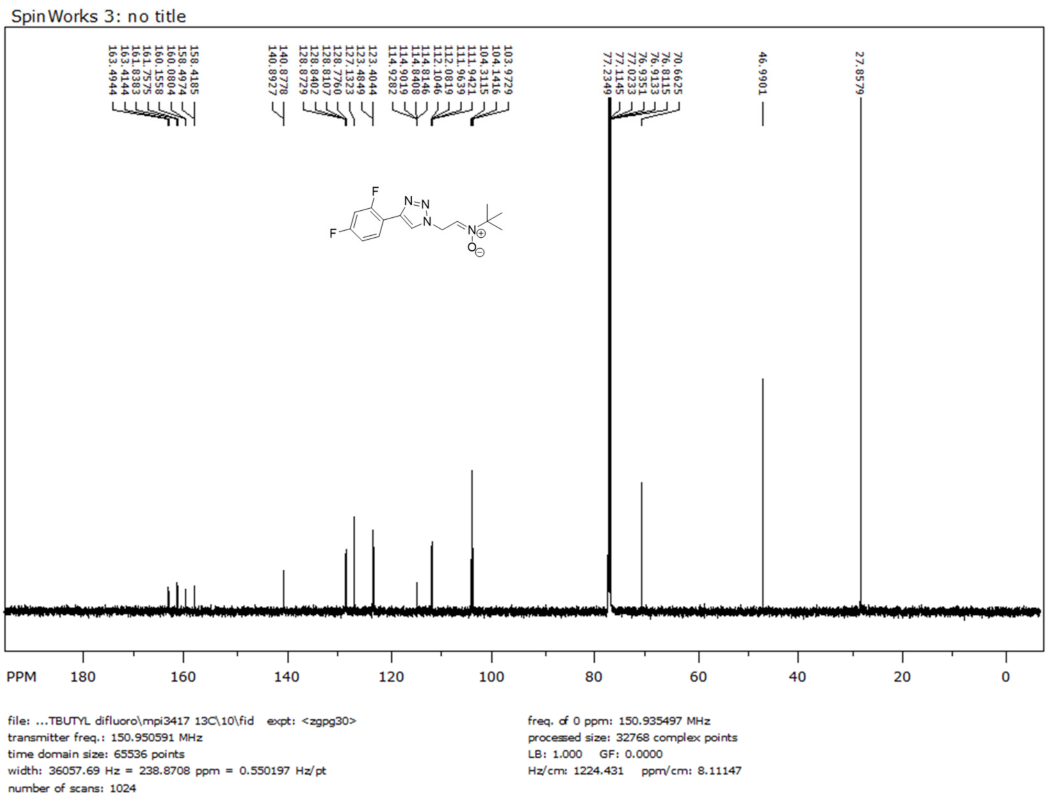

Figure S22: ^{13}C NMR Spectrum for **9e** in $CDCl_3$

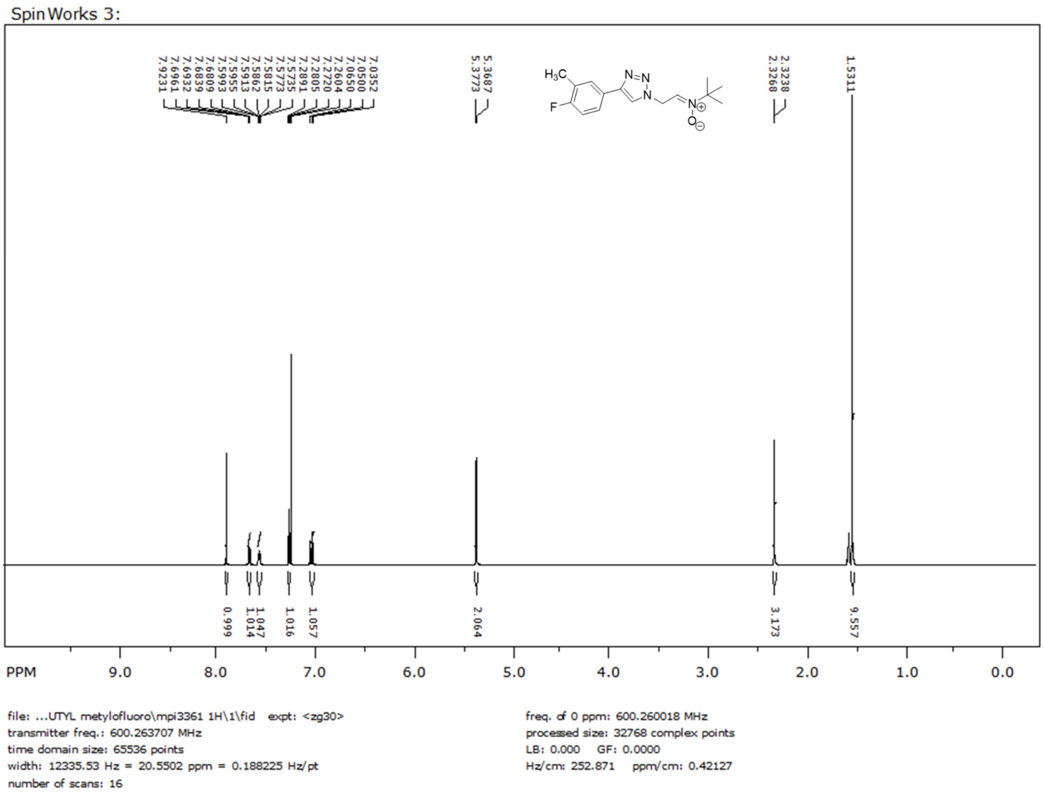

Figure S23: ^{1}H NMR Spectrum for **9f** in D_2O

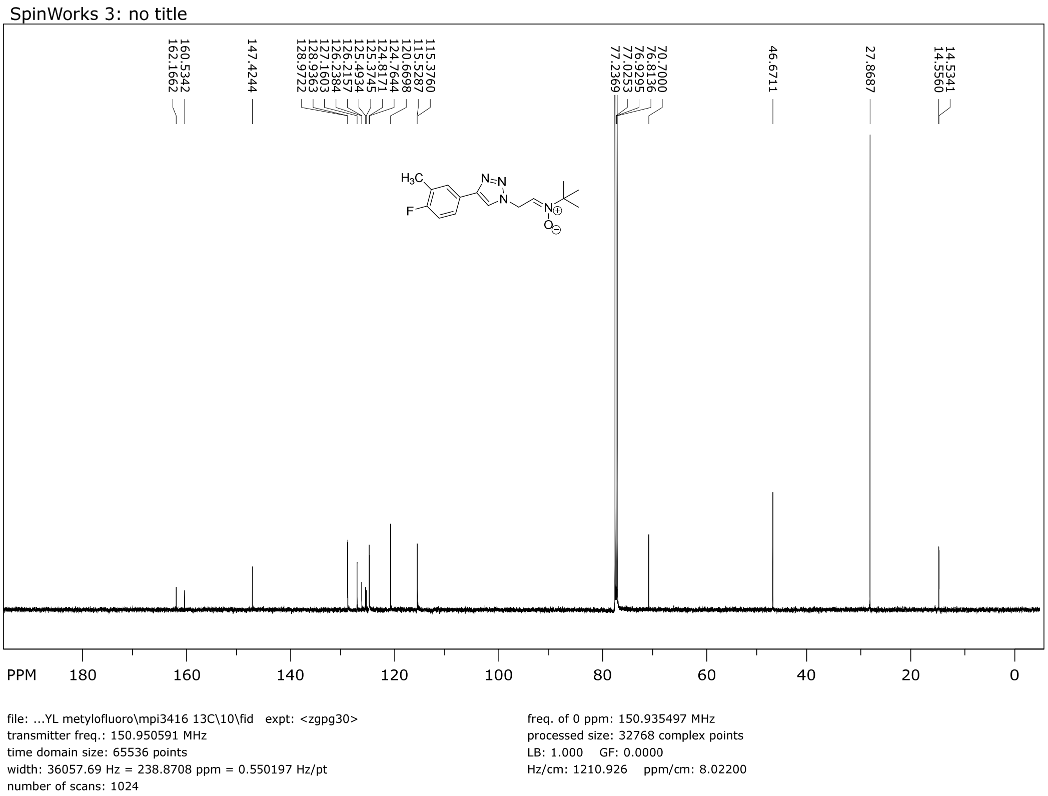

Figure S24: ^{13}C NMR Spectrum for **9f** in $CDCl_3$
